# MR.RGM: an R package for fitting Bayesian multivariate bidirectional Mendelian randomization networks

**DOI:** 10.1093/bioinformatics/btaf130

**Published:** 2025-03-24

**Authors:** Bitan Sarkar, Yang Ni

**Affiliations:** De partment of Statistics, Texas A&M University, College Station, TX 77843, United States; De partment of Statistics, Texas A&M University, College Station, TX 77843, United States; Department of Statistics and Data Sciences, The University of Texas, Austin, TX 78705, United States

## Abstract

**Motivation:**

Mendelian randomization (MR) infers causal relationships between exposures and outcomes using genetic variants as instrumental variables. Typically, MR considers only a pair of exposure and outcome at a time, limiting its capability of capturing the entire causal network. We overcome this limitation by developing **MR.RGM** (Mendelian randomization via reciprocal graphical model), a fast R-package that implements the Bayesian reciprocal graphical model and enables practitioners to construct holistic causal networks with possibly cyclic/reciprocal causation and proper uncertainty quantifications, offering a comprehensive understanding of complex biological systems and their interconnections.

**Results:**

We developed **MR.RGM**, an open-source R package that applies bidirectional MR using a network-based strategy, enabling the exploration of causal relationships among multiple variables in complex biological systems. **MR.RGM** holds the promise of unveiling intricate interactions and advancing our understanding of genetic networks, disease risks, and phenotypic complexities.

**Availability and implementation:**

**MR.RGM** is available at CRAN (https://CRAN.R-project.org/package=MR.RGM, DOI: 10.32614/CRAN.package.MR.RGM) and https://github.com/bitansa/MR.RGM.

## 1 Introduction

Mendelian randomization (MR) is a vital tool in genetic and epidemiological studies, emulating randomized control trials to elucidate causal relationships between exposures and outcomes. MR utilizes genetic variants as instrumental variables to mitigate confounding biases in observational studies. While traditional MR focuses on individual exposures and specific outcomes, the challenge arises in exploring multifaceted causal relationships involving multiple outcomes in complex biological systems.

A comprehensive review of existing R packages for MR reveals a range of tools with distinct functionalities. The package **mr.pivw** (Xu 2022) implements the penalized inverse-variance weighted (pIVW) estimator, designed to estimate causal effects using summary-level data from genome-wide association studies. **mr.raps** (Zhao 2018) utilizes the Robust Adjusted Profile Score (RAPS) method for two-sample MR, enhancing causal inference through summary statistics. **PPMR** (Yuan and Zhou 2019) excels in efficient two-sample MR analysis, addressing issues related to correlated instruments and horizontal pleiotropy.

**OneSampleMR** (Palmer *et al.* 2023) is tailored for one-sample MR and instrumental variable analyses, leveraging individual-level data for detailed analyses. **MRPC** (Badsha and Fu 2022) applies principal component analysis within the MR framework, facilitating multivariate MR by summarizing complex relationships into principal components. **MendelianRandomization** (Burgess and Yavorska 2024) supports both univariate and multivariate approaches using summary data, providing a versatile tool for complex MR analyses.

Recent advancements include **TwoSampleMR** (Hemani *et al.* 2018), designed for robust two-sample MR analyses, and **MVMR** (Sanderson *et al.* 2021), which efficiently handles multivariate MR by modeling multiple outcomes and exposures. **mrbayes** (Uche-Ikonne *et al.* 2021) provides Bayesian implementation of multivariate MR based on either the inverse variance weighted model (Thompson 2013) or the Egger regression model (Bowden *et al.* 2015), enabling uncertainty quantification through MCMC sampling. **MrDAG** (Bottolo and Zuber 2024, Zuber *et al.* 2024) uses directed acyclic graphs to elucidate causal structures, focusing on causal inference within a Bayesian network framework.

Each package contributes significantly to MR research with distinct functionalities tailored to different data scenarios. However, common limitations include challenges in uncertainty quantification, restrictions to specific data types, constraints to bivariate analyses, and limitation in application to bidirectional MR. For details, refer to Table 1.

**Table 1.** “✓” indicates that a package is capable of performing a specific Sutask, while “✗” denotes that the package is not equipped to handle the task.^a^

R package	Multivariate MR	Summary level data	Uncertainty quantification	Bidirectionality
mr.pivw	✗	✓	✓	✗
mr.raps	✓	✓	✓	✗
PPMR	✓	✓	✓	✗
MRPC	✓	✓	✓	✗
OneSampleMR	✗	✗	✓	✗
Mendelian randomization	✓	✓	✓	✗
TwoSampleMR	✓	✓	✓	✗
MVMR	✓	✓	✓	✗
mrbayes	✓	✓	✓	✗
MrDAG	✓	✓	✓	✗
MR.RGM	✓	✓	✓	✓

a Note that even if a package is not designed specifically for bidirectional MR (indicated by “✗”), it can be in principle repeatedly applied to infer bi-directional causation.

This work introduces **MR.RGM** (Sarkar and Ni 2024) (for modeling details, refer to Supplementary Materials, Supplementary Section S1), a new package for multivariate bidirectional MR based on reciprocal graphical model (Ni *et al.* 2018). **MR.RGM** uses a Bayesian framework and a network-based strategy, surpassing traditional MR approaches. While conventional methods focus on isolated variables, **MR.RGM** constructs comprehensive causal networks possibly with feedback loops, particularly adept at capturing intricate relationships within complex biological systems. **MR.RGM** supports individual-level data and two types of summary-level data, leveraging C++ for backend computations and the Woodbury matrix identity for efficiency (refer to Supplementary Materials, Supplementary Sections S6 and S7). Its Bayesian nature enables natural uncertainty quantification.

## 2 Methods

The R package **MR.RGM** has two key functions: **RGM**, dedicated to building causal graphs and estimating causal effects, and **NetworkMotif**, designed for uncertainty quantification, providing posterior probabilities for specific network motifs. First, we delve into the functionality of the **RGM** function, followed by a discussion of the **NetworkMotif** function.

For detailed implementation and usage examples, see Supplementary Section S5 of the Supplementary Materials.

### 2.1 Function 1: RGM

#### 2.1.1 Three input formats

**RGM** supports three input formats, catering to various data availability scenarios.

#### 2.1.2 Individual-level data input

Researchers input ***X*** and ***Y*** matrices. ***X*** signifies the instrument data matrix, where columns represent instruments (e.g. SNPs), and rows represent observations. ***Y*** denotes the response data matrix, with columns representing response variables (e.g. protein, gene, or phenotype), and rows corresponding to observations.

#### 2.1.3 Summary-level data input

Recognizing that individual-level data, especially SNPs or disease status, can be sensitive and not always readily available, **RGM** provides support for two types of summary-level data input. The first type involves specifying ***Syy***, ***Syx***, and ***Sxx*** matrices. ***Syy*** represents the covariance matrix of the response variables, ***Syx*** signifies the covariance matrix between the responses and instruments, and ***Sxx*** denotes the covariance matrix of the instrument variables. **RGM** would output the exact same solution as that based on individual-level data. For the calculation of the likelihood using summary-level data, refer to Supplementary Materials, Supplementary Section S9.

#### 2.1.4 Handling difficult cross-correlations

In situations where obtaining cross-correlations among response variables (***Syy***) is challenging, for instance, when two response variables were never measured in the same dataset, **RGM** can still be used and will output an approximate solution. In this scenario, researchers can specify ***Sxx***, ***Beta***, and ***SigmaHat*** matrices as input. ***Beta*** and ***SigmaHat*** matrices respectively contain regression coefficients and mean square errors for the regression of a response variable on its corresponding instrument, where the regression is performed without including an intercept term. Each row of ***Beta*** and ***SigmaHat*** matrices corresponds to a response variable, and each column corresponds to an instrument. For the detailed derivation, please refer to Supplementary Sections S10 and S11 of the Supplementary Materials.

#### 2.1.5 Essential input parameters

Users need to specify two input parameters: ***D*** and ***n***. The matrix ***D*** is binary with dimensions *p* × *k*, where *p* is the number of response variables and *k* is the number of instrumental variables. Each row represents a response variable, and each column represents an instrumental variable. A 1 in ***D***[*i*, *j*] indicates that instrumental variable *j* affects response variable *i*. For identifiability (refer to Supplementary Materials, Supplementary Section S4), each response must be influenced by at least one instrumental variable that affects only that response, meaning there must be at least one unique entry of 1 in each row, where the corresponding column contains 0 in all other rows. If this condition is violated, the algorithm will throw an error when using ***Syy***, ***Beta***, and ***SigmHat***, but will issue a warning and proceed when using ***X***, ***Y***, or ***Syy***, ***Syx***, ***Sxx***. The parameter ***n*** is the sample size (only needed for summary-level data).

#### 2.1.6 Customizable parameters

**RGM** offers customizable analysis through user-defined parameters. Users can specify the number of MCMC sampling iterations (***nIter***) with a default of 10 000, set the number of discarded samples (***nBurnin***) with a default of 2000, and define the thinning of posterior samples using the ***Thin*** parameter (default value: 1). Additionally, users can choose between two graph structure priors: ***Spike and Slab*** or ***Threshold***, with the default being ***Spike and Slab***. For details of these priors, refer to Supplementary Materials, Supplementary Section S2.

Users also have the option to customize the model hyperparameters aRho, ***bRho***, ***nu1***, ***aPsi***, ***bPsi***, ***nu2***, ***aSigma***, ***bSigma***, ***PropVarA***and ***PropVarB***, although relatively robust default values are provided for ease of use. For details, see Supplementary Section S3 of the Supplementary Materials.

#### 2.1.7 Outputs

The main outputs of **RGM** encompass causal effect estimates (***AEst*** and ***BEst***) among response variables and between instruments and responses. Additionally, **RGM** outputs binary adjacency matrices (***zAEst*** and ***zBEst***) that depict graph structures, derived by thresholding the posterior probability matrices (***GammaEst*** and ***PhiEst*** respectively) at 0.5. The thresholding process may result in some entries of ***zAEst*** being zero even when the corresponding entries in ***AEst*** are non-zero. This discrepancy arises because ***AEst*** represents the posterior mean of causal effects, while ***zAEst*** reflects whether the inclusion probability for an edge exceeds the threshold (0.5). For practical use, multiplying ***AEst*** by ***zAEst*** elementwise can help account for edge inclusion probabilities, effectively making causal effects zero where inclusion probabilities are very small. **RGM** return an output **Graph**, which is a graph object that incorporates these ideas, encoding both causal directions and causal effect sizes. Plotting this graph provides a clear visualization of the causal network among responses. **RGM** further provides posterior samples of the adjacency matrix between response variables (***GammaPst***), an input for the **NetworkMotif** function to quantify uncertainty for a specific network motif. Other outputs comprise of ***A0Est***, ***B0Est***, ***GammaEst***, ***TauEst***, ***RhoEst***, ***PhiEst***, ***EtaEst***, ***PsiEst***, ***tAEst***, ***tBEst***, ***SigmaEst***, ***AccptA***, ***AccptB***, ***AccpttA***, ***AccpttB***, and ***LLPst***. All these outputs represent posterior means except ***LLPst***, which consists of posterior samples; see Supplementary Section S3 of the Supplementary Materials for details. Next, we will describe the **NetworkMotif** function.

### 2.2 Function 2: NetworkMotif

#### 2.2.1 Inputs

The **NetworkMotif** function requires two input variables: ***Gamma*** and ***GammaPst***. ***Gamma*** represents a specific adjacency matrix corresponding to the network motif of the response variables for which uncertainty quantification is desired. ***GammaPst*** consists of the posterior samples of the adjacency matrix obtained from the **RGM** function.

#### 2.2.2 Output

The output of the **NetworkMotif** function includes the uncertainty quantification (i.e. posterior probability) of the specified network motif. For details, please refer to Supplementary Section S3 of the Supplementary Materials.

## 3 Simulation results

**Figure 1.**
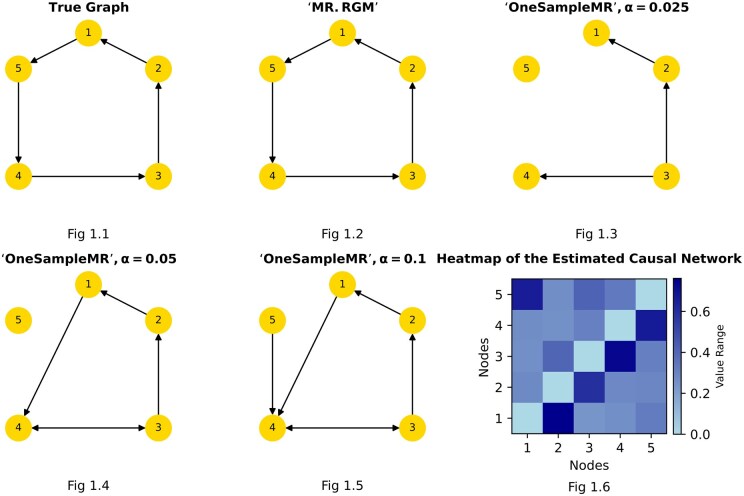
Figure 1.1 represents the true graph. Figure 1.2 illustrates the causal network estimated by **MR.RGM** with the Spike and Slab prior. Figure 1.3, 1.4, and 1.5 depict the causal graph estimated by **OneSampleMR** with different alpha values. Figure 1.6 displays the heatmap of the causal graph created by **MR.RGM**. In the heatmap, each entry corresponds to node *i* in the horizontal row and node *j* in the vertical row, indicating the posterior probability of the causal effect of node *j* on node *i*. Lighter colors signify lower probabilities of any causal effect.

Utilizing the mathematical model *y* = A*y* + B*x* + *e*, we conducted a comprehensive assessment of our algorithm's performance. By generating *x* and *e* from a normal distribution, we simulated various scenarios to gauge the effectiveness of our approach. We compared our package with **OneSampleMR**, a recent and advanced R package for MR, based on true positive rate (TPR), false positive rate (FPR), false discovery rate (FDR), Matthew's correlation coefficient (MCC), and area under the receiver operating characteristic curve (AUC) at different sample sizes (10k, 30k, 50k), network sizes (5, 10), sparsity of A (25%, 50%), and effect sizes (0.1, −0.1). We adjust the standard deviation of the noise to achieve different variance explained levels (1%, 3%, 5%, and 10%). B is set to be the identity matrix. The detailed results are presented in the Supplementary Materials, Supplementary Section S12 and Supplementary Tables S2–S5. Notably, our approach consistently yielded equivalent results when applied to both individual-level data and summary-level data, validating its versatility and reliability across different data availability scenarios. As an example of comparison (Fig. 1), it is evident that the networks produced by our method, exhibit a substantial improvement in accuracy compared to those generated by the package **OneSampleMR**. For further details on the selection of instrumental variables in real-world data and the application of these methods to GTEx (GTEx Consortium 2020) V7 data, please refer to Supplementary Materials, Supplementary Section S13. In addition, we evaluated **MR.RGM**'s sensitivity to the assumption of normal errors by comparing performance across various error distributions, including normal, t-distributions, and Laplace. The results, indicating that **MR.RGM** is not sensitive to the normal error assumption, are detailed in Supplementary Materials, Supplementary Section S8 and Supplementary Table S1.

## 4 Conclusion

This paper introduces **MR.RGM**, an R package for exploring causal relationships in complex biological systems. **MR.RGM** constructs comprehensive causal graphs possibly with feedback loops, accommodating both individual and summary-level data. Its versatility, adaptability to diverse data formats, and network-based strategy enhance causal inference. **MR.RGM**'s Bayesian approach enables uncertainty quantification and holds the promise to advance genetics and epidemiology research by uncovering complex biological phenomena.

## Supplementary Material

btaf130_Supplementary_Data

## Data Availability

The GTEx data were downloaded from the GTEx Consortium.
