# Supplementary material for: MR.RGM: an R package for fitting Bayesian multivariate bidirectional Mendelian randomization networks
Source: Bioinformatics. 2025 Mar 24;41(4):btaf130. doi: 10.1093/bioinformatics/btaf130 (PMC11992333; doi:10.1093/bioinformatics/btaf130)
Supplement: btaf130_Supplementary_Data [file btaf130_supplementary_data.pdf]

SUPPLEMENTARY MATERIAL MR.RGM: An R Package for Fitting Bayesian Multivariate Bidirectional Mendelian Randomization Networks

Bitan Sarkar[1] and Yang Ni[1]

[1] Department of Statistics, Texas A&M University, 3143, College Station, TX 77843, USA

1 Model

Let $\mathbf{Y_i} = (Y_{i1}, \cdots, Y_{ip})^T$ denotes the expressions for response variables $1, \cdots, p$, let $\mathbf{X_i} = (X_{i1}, \cdots, X_{ik})^T$ be the set of measurements for instrument variables $1, \cdots, k$ and for $i = 1, \ldots, n$. We have the following model:

$$\begin{aligned} &\mathbf{Y_i} = \mathbf{AY_i} + \mathbf{BX_i} + \mathbf{E_i}, \quad \mathbf{E_i} \sim N(0, \mathbf{\Sigma}) \\ \Longrightarrow\ &(\mathbf{I_p} - \mathbf{A})\mathbf{Y_i} = \mathbf{BX_i} + \mathbf{E_i} \\ \Longrightarrow\ &\mathbf{Y_i} = (\mathbf{I_p} - \mathbf{A})^{-1}\mathbf{BX_i} + (\mathbf{I_p} - \mathbf{A})^{-1}\mathbf{E_i} \end{aligned} \tag{1.1}$$

where $\mathbf{A} = (a_{ij}) \in \mathbf{R}^{p\times p}$ with zeroes on the diagonal, denotes the causal effects or strengths between the response variables, $\mathbf{B} = (b_{il}) \in \mathbf{R}^{p\times k}$ denotes the causal effects or strengths of the instrument variables on the response variables, $\mathbf{E_i} = (\epsilon_{1i}, \cdots, \epsilon_{pi})^T \sim N_p(0, \mathbf{\Sigma})$ and $\mathbf{E_i}$ and $\mathbf{X_i}$ are independent for $i = 1, \ldots, n$. $\mathbf{I_p}$ denotes a $p \times p$ identity matrix. We assume that $\mathbf{\Sigma}$ is diagonal i.e. $\mathbf{\Sigma} = \text{diag}(\sigma_1, \cdots, \sigma_p)$, i.e. the residuals are independent. Thus model (1.1) can be equivalently expressed as:

$$\mathbf{Y_i}|\mathbf{X_i} \sim N_p\{(\mathbf{I}_p - \mathbf{A})^{-1}\mathbf{BX_i}, (\mathbf{I}_p - \mathbf{A})^{-1}\mathbf{\Sigma}(\mathbf{I}_p - \mathbf{A})^{-T}\} \tag{1.2}$$

2 Prior Specification

MR.RGM [1, 2] offers the capability to estimate graph structures both among response variables and between response and instrument variables. This estimation process accommodates two distinct prior assumptions: "**Threshold**" and "**Spike and Slab**" priors, which users can specify through the "**prior**" parameter in **RGM**. The prior assumptions significantly influence the parameter settings and the full conditionals employed during the MCMC sampling procedure. We have the following model:

$$\mathbf{Y_i} = \mathbf{AY_i} + \mathbf{BX_i} + \mathbf{E_i},\ i = 1, \ldots, n, \tag{2.1}$$

where $\mathbf{A} = (a_{ij}) \in \mathbf{R}^{p\times p}$ with zeroes on the diagonal, denotes the causal effect or strength between the response variable, $\mathbf{B} = (b_{il}) \in \mathbf{R}^{p\times k}$ denotes the causal effects or strengths of instrument variables on response variables, $\mathbf{E_i} = (\epsilon_{1i}, \cdots, \epsilon_{pi})^T \sim N_p(0, \mathbf{\Sigma})$ and $\mathbf{E_i}$ and $\mathbf{X_i}$ are independent,

for $i = 1, \ldots, n$. We assume that $\mathbf{\Sigma}$ is diagonal, i.e. $\mathbf{\Sigma} = \mathrm{diag}(\sigma_1, \cdots, \sigma_p)$, i.e. the residuals are independent. Here's an overview of the prior assumptions on the model parameters and their impact on the analysis:

2.1 Threshold Prior

We have the following prior assumptions in this case:

$$
\begin{aligned}
\tilde{a}_{ij} &\sim N(0, \tau_{ij}), \quad (i = 1, \ldots, p;\ j = 1, \ldots, p) \\
a_{ij} &= \tilde{a}_{ij}\mathbf{I}(|\tilde{a}_{ij}| > t_A), \quad (i = 1, \ldots, p;\ j = 1, \ldots, p) \\
t_A &\sim Uniform(0, 1), \\
\sqrt{\tau_{ij}} &\sim C^+(0, 1), \quad (i = 1, \ldots, p;\ j = 1, \ldots, p) \\
\\
\tilde{b}_{il} &\sim N(0, \eta_{il}), \quad (i = 1, \ldots, p;\ l = 1, \ldots, k) \\
b_{il} &= \tilde{b}_{il}\mathbf{I}(|\tilde{b}_{il}| > t_B), \quad (i = 1, \ldots, p;\ l = 1, \ldots, k) \\
t_B &\sim Uniform(0, 1), \\
\sqrt{\eta_{il}} &\sim C^+(0, 1), \quad (i = 1, \ldots, p;\ l = 1, \ldots, k) \\
\\
\sigma_i &\sim IG(a_\sigma, b_\sigma), \quad (i = 1, \ldots, p)
\end{aligned}
\tag{2.2}
$$

where $C^+(0, 1)$ denotes standard half-cauchy distribution and $x \sim C^+(0, 1)$ implies the following:

$$
x^2 | a \sim IG(1/2, 1/a) \quad \text{and} \quad a \sim IG(1/2, 1).
$$

Posterior inference under Threshold prior is implemented by the following MCMC posterior simulation.

1. Update η_{il} by a Gibbs transition probability. Draw $\epsilon \sim IG(1, 1 + 1/\eta_{il})$ and then draw $\eta_{il} \sim IG(1, \tilde{b}_{il}^2/2 + 1/\epsilon)$.

2. Update $\tilde{b}_{il}$ and b_{il} by a random walk Metropolis-Hastings (M-H) transition probability. Draw $\tilde{b}_{il}^* \sim N(\tilde{b}_{il}, \mathbf{PropVarB})$ and create $\mathbf{B}^*$ from $\mathbf{B}$ by substituting b_{il} by b_{il}^* where $b_{il}^* = \tilde{b}_{il}^*\mathbf{I}(|\tilde{b}_{il}^*| > t_B)$. Accept $\tilde{b}_{il}^*$ and b_{il}^* with probability $\min(\alpha, 1)$ where,

$$
\alpha = \frac{p(\mathbf{Y}|\mathbf{X}, \mathbf{A}, \mathbf{B}^*, \mathbf{\Sigma})p(\tilde{b}_{il}^*|\eta_{il})}{p(\mathbf{Y}|\mathbf{X}, \mathbf{A}, \mathbf{B}, \mathbf{\Sigma})p(\tilde{b}_{il}|\eta_{il})}.
$$

3. Update ϕ_{il} based on the value of b_{il}. Make $\phi_{il} = 1$ if $b_{il} \neq 0$, else, make $\phi_{il} = 0$.

4. Update t_B by an M-H transition probability with a truncated normal proposal. Draw $t_B^* \sim TN(t_B, 0.01, 0, 1)$ where $TN(t_B, 0.01, 0, 1)$ denotes a normal distribution with mean t_B, variance 0.01 and restricted to the interval $(0, 1)$ and accept it with probability $\min(\alpha, 1)$ where,

$$
\alpha = \frac{p(\mathbf{Y}|\mathbf{X}, \mathbf{A}, \mathbf{B}^*, \mathbf{\Sigma})TN(t_B|t_B^*, 0.01, 0, 1)}{p(\mathbf{Y}|\mathbf{X}, \mathbf{A}, \mathbf{B}, \mathbf{\Sigma})TN(t_B^*|t_B, 0.01, 0, 1)}.
$$

$\mathbf{B}^*$ is obtained by thresholding $\tilde{\mathbf{B}}$ at t_B^*.

5. Update σ_j by a Gibbs transition probability. Draw $\sigma_j \sim IG(a_\sigma + n/2, b_\sigma + SSE_j/2)$ where,

$$\begin{aligned}SSE_j =& n \times (\mathbf{I_p} - \mathbf{A})_{(\mathbf{j},.)} \times \mathbf{Syy} \times (\mathbf{I_p} - \mathbf{A})_{(\mathbf{j},.)}{}^T - 2 \times n \times (\mathbf{I_p} - \mathbf{A})_{(\mathbf{j},.)} \times \mathbf{Syx} \times \mathbf{B}_{(\mathbf{j},.)}{}^T \\ &+ n \times \mathbf{B}_{(\mathbf{j},.)} \times \mathbf{Sxx} \times \mathbf{B}_{(\mathbf{j},.)}{}^T.\end{aligned}$$

$\mathbf{I_p}$ denotes a $p \times p$ identity matrix, $(\mathbf{I_p} - \mathbf{A})_{(\mathbf{j},.)}$ denotes jth row of $(\mathbf{I_p} - \mathbf{A})$ and $\mathbf{B}_{(\mathbf{j},.)}$ denotes jth row of $\mathbf{B}$.

6. Update τ_{ij} by a Gibbs transition probability. Draw $\epsilon \sim IG(1, 1 + 1/\tau_{ij})$ and then draw $\tau_{ij} \sim IG(1, \tilde{a}_{ij}^2/2 + 1/\epsilon)$.

7. Update $\tilde{a}_{ij}$ and a_{ij} by a random walk Metropolis-Hastings (M-H) transition probability. Draw $\tilde{a}_{ij}^* \sim N(\tilde{a}_{ij}, \mathbf{PropVarA})$ and create A^* from A by substituting a_{ij} by a_{ij}^* where $a_{ij}^* = \tilde{a}_{ij}^* \mathbf{I}(|\tilde{a}_{ij}^*| > t_A)$. Accept $\tilde{a}_{ij}^*$ and a_{ij}^* with probability $\min(\alpha, 1)$ where,

$$\alpha = \frac{p(\mathbf{Y}|\mathbf{X}, \mathbf{A}^*, \mathbf{B}, \mathbf{\Sigma}) p(\tilde{a}_{ij}^*|\tau_{ij})}{p(\mathbf{Y}|\mathbf{X}, \mathbf{A}, \mathbf{B}, \mathbf{\Sigma}) p(\tilde{a}_{ij}|\tau_{ij})}.$$

8. Update γ_{ij} based on the value of a_{ij}. Make $\gamma_{ij} = 1$ if $a_{ij} \neq 0$, else, make $\gamma_{ij} = 0$.

9. Update t_A by an M-H transition probability with a truncated normal proposal. Draw $t_A^* \sim TN(t_A, 0.01, 0, 1)$ where $TN(t_A, 0.01, 0, 1)$ denotes a normal distribution with mean t_A, variance 0.01 and restricted to the interval $(0, 1)$ and accept it with probability $\min(\alpha, 1)$ where,

$$\alpha = \frac{p(\mathbf{Y}|\mathbf{X}, \mathbf{A}^*, \mathbf{B}, \mathbf{\Sigma}) TN(t_A|t_A^*, 0.01, 0, 1)}{p(\mathbf{Y}|\mathbf{X}, \mathbf{A}, \mathbf{B}, \mathbf{\Sigma}) TN(t_A^*|t_A, 0.01, 0, 1)}.$$

$\mathbf{A}^*$ is obtained by thresholding $\tilde{\mathbf{A}}$ at t_A^*.

2.2 Spike and Slab Prior

We have the following prior assumptions in this case:

$$\begin{aligned}
a_{ij} &\sim \gamma_{ij} N(0, \tau_{ij}) + (1 - \gamma_{ij}) N(0, \nu_1 \times \tau_{ij}), \quad (i = 1, \dots, p; \, j = 1, \dots, p) \\
\gamma_{ij} &\sim Ber(\rho_{ij}), \quad (i = 1, \dots, p; \, j = 1, \dots, p) \\
\rho_{ij} &\sim Beta(a_\rho, b_\rho), \quad (i = 1, \dots, p; \, j = 1, \dots, p) \\
\sqrt{\tau_{ij}} &\sim C^+(0, 1), \quad (i = 1, \dots, p; \, j = 1, \dots, p) \\
\\
b_{il} &\sim \phi_{il} N(0, \eta_{il}) + (1 - \phi_{il}) N(0, \nu_2 \times \eta_{il}), \quad (i = 1, \dots, p; \, l = 1, \dots, k) \qquad (2.3) \\
\phi_{il} &\sim Ber(\psi_{il}), \quad (i = 1, \dots, p; \, l = 1, \dots, k) \\
\psi_{il} &\sim Beta(a_\psi, b_\psi), \quad (i = 1, \dots, p; \, l = 1, \dots, k) \\
\sqrt{\eta_{il}} &\sim C^+(0, 1), \quad (i = 1, \dots, p; \, l = 1, \dots, k) \\
\\
\sigma_i &\sim IG(a_\sigma, b_\sigma), \quad (i = 1, \dots, p)
\end{aligned}$$

Posterior inference under Spike and Slab prior is implemented by the following MCMC posterior simulation.

1. Update ψ_{il} by a Gibbs transition probability. Draw $\psi_{il} \sim Beta(\phi_{il} + a_\psi, 1 - \phi_{il} + b_\psi)$.

2. Update η_{il} by a Gibbs transition probability. Draw $\epsilon \sim IG(1, 1 + 1/\eta_{il})$ and then draw $\eta_{il} \sim IG(1, b_{il}^2/2 + 1/\epsilon)$ (if $\phi_{il} = 1$) or draw $\eta_{il} \sim IG(1, b_{il}^2/(2 \times \nu_2) + 1/\epsilon)$ (if $\phi_{il} = 0$).

3. Update ϕ_{il} by a Gibbs transition probability. Draw $\phi_{il} \sim Ber(p)$ where,

$$p = \frac{\exp\left(-b_{il}^2/(2 \times \eta_{il})\right) \times \psi_{il}}{\exp\left(-b_{il}^2/(2 \times \eta_{il})\right) \times \psi_{il} + \exp\left(-b_{il}^2/(2 \times \nu_2 \times \eta_{il})\right) \times (1 - \psi_{il})/\sqrt{\nu_2}}.$$

4. Update b_{il} by a random walk Metropolis-Hastings (M-H) transition probability. Draw $b_{il}^* \sim N(b_{il}, \mathbf{PropVarB})$ and create $\mathbf{B}^*$ from $\mathbf{B}$ by substituting b_{il} by b_{il}^*. Accept b_{il}^* with probability $\min(\alpha, 1)$ where,

$$\alpha = \frac{p(\mathbf{Y}|\mathbf{X}, \mathbf{A}, \mathbf{B}^*, \mathbf{\Sigma})p(b_{il}^*|\phi_{il}, \eta_{il}, \nu_2)}{p(\mathbf{Y}|\mathbf{X}, \mathbf{A}, \mathbf{B}, \mathbf{\Sigma})p(b_{il}|\phi_{il}, \eta_{il}, \nu_2)}.$$

5. Update σ_j by a Gibbs transition probability. Draw $\sigma_j \sim IG(a_\sigma + n/2, b_\sigma + SSE_j/2)$ where,

$$\begin{aligned} SSE_j =& n \times (\mathbf{I_p} - \mathbf{A})_{(\mathbf{j},.)} \times \mathbf{Syy} \times (\mathbf{I_p} - \mathbf{A})_{(\mathbf{j},.)}^{\mathbf{T}} - 2 \times n \times (\mathbf{I_p} - \mathbf{A})_{(\mathbf{j},.)} \times \mathbf{Syx} \times \mathbf{B}_{(\mathbf{j},.)}^{\mathbf{T}} \\ &+ n \times \mathbf{B}_{(\mathbf{j},.)} \times \mathbf{Sxx} \times \mathbf{B}_{(\mathbf{j},.)}^{\mathbf{T}}. \end{aligned}$$

$\mathbf{I_p}$ denotes a $p \times p$ identity matrix, $(\mathbf{I_p} - \mathbf{A})_{(\mathbf{j},.)}$ denotes jth row of $(\mathbf{I_p} - \mathbf{A})$ and $\mathbf{B}_{(\mathbf{j},.)}$ denotes jth row of $\mathbf{B}$.

6. Update ρ_{ij} by a Gibbs transition probability. Draw $\rho_{ij} \sim Beta(\gamma_{ij} + a_\rho, 1 - \gamma_{ij} + b_\rho)$.

7. Update τ_{ij} by a Gibbs transition probability. Draw $\epsilon \sim IG(1, 1 + 1/\tau_{ij})$ and then draw $\tau_{ij} \sim IG(1, a_{ij}^2/2 + 1/\epsilon)$ (if $\gamma_{ij} = 1$) or draw $\tau_{ij} \sim IG(1, a_{ij}^2/(2 \times \nu_1) + 1/\epsilon)$ (if $\gamma_{ij} = 0$).

8. Update γ_{ij} by a Gibbs transition probability. Draw $\gamma_{ij} \sim Ber(p)$ where

$$p = \frac{\exp\left(-a_{ij}^2/(2 \times \tau_{ij})\right) \times \rho_{ij}}{\exp\left(-a_{ij}^2/(2 \times \tau_{ij})\right) \times \rho_{ij} + \exp\left(-a_{ij}^2/(2 \times \nu_1 \times \tau_{ij})\right) \times (1 - \rho_{ij})/\sqrt{\nu_1}}.$$

9. Update a_{ij} by a random walk Metropolis-Hastings (M-H) transition probability. Draw $a_{ij}^* \sim N(a_{ij}, \mathbf{PropVarA})$ and create $\mathbf{A}^*$ from $\mathbf{A}$ by substituting a_{ij} by a_{ij}^*. Accept a_{ij}^* with probability $\min(\alpha, 1)$ where,

$$\alpha = \frac{p(\mathbf{Y}|\mathbf{X}, \mathbf{A}^*, \mathbf{B}, \mathbf{\Sigma})p(a_{ij}^*|\gamma_{ij}, \tau_{ij}, \nu_1)}{p(\mathbf{Y}|\mathbf{X}, \mathbf{A}, \mathbf{B}, \mathbf{\Sigma})p(a_{ij}|\gamma_{ij}, \tau_{ij}, \nu_1)}.$$

3 Available Functions

MR.RGM provides two user-accessible functions: **RGM** and **NetworkMotif**. The primary objective of the **RGM** function is to establish causal networks among response variables and between response and instrument variables, while **NetworkMotif** focuses on quantifying uncertainty for a given network structure among the response variables. Detailed input and output structures for these functions, along with usage guidelines, are provided in the following subsections.

3.1 RGM

3.1.1 Inputs

We will now specify the inputs for **RGM** and give brief descriptions about them.

- **X**: **X** represents the instrument data matrix, which is $n \times k$, where each column corresponds to an instrument (e.g. DNA or SNPs), and each row represents a particular observation. The default value is set to **NULL**.

- **Y**: **Y** represents the response data matrix, which is $n \times p$, where each column corresponds to a response (e.g. protein, gene or disease data), and each row represents a particular observation. The default value is set to **NULL**.

- **Syy**: **Syy** represents the covariance matrix of the response variables. It is a matrix of dimensions $p \times p$. Here, "p" signifies the number of response variables. This matrix is derived through the operation $(\mathbf{Y}^T \times \mathbf{Y})/n$, where "$\mathbf{Y}$" denotes the response data matrix and "n" stands for the total number of observations.

- **Syx**: **Syx** represents the covariance matrix between the responses and the instruments. It is a matrix of dimensions $p \times k$. Here, "p" signifies the number of response variables and "k" represents the number of instrument variables. This matrix is derived through the operation $(\mathbf{Y}^T \times \mathbf{X})/n$, where "$\mathbf{Y}$" denotes the response data matrix, "$\mathbf{X}$" denotes the instrument data matrix and "n" stands for the total number of observations.

- **Sxx**: **Sxx** represents the covariance matrix of the instrument variables. It is a matrix of dimensions $k \times k$. Here, "k" signifies the number of instrument variables. This matrix is derived through the operation $(\mathbf{X}^T \times \mathbf{X})/n$, where "$\mathbf{X}$" denotes the instrument data matrix and "n" stands for the total number of observations.

- **Beta**: **Beta** is a matrix of dimensions $p \times k$. In this matrix, each row corresponds to a specific response variable, and each column pertains to a distinct instrument variable. Each entry within the matrix represents the regression coefficient of the individual response variable on the specific instrument variable, where the regression is performed without including an intercept term.

- **SigmaHat**: **SigmaHat** is a matrix of dimensions $p \times k$. In this matrix, each row corresponds to a specific response variable, and each column pertains to an individual instrument variable. Each entry in this matrix represents the mean square error associated with regressing the particular response on the specific instrument variable, where the regression is performed without including an intercept term.

- **D**: **D** is a binary indicator matrix of dimension $p \times k$, where p represents the number of response variables, and k represents the total number of instrumental variables. Each row of **D** corresponds to a response variable, and each column corresponds to an instrumental variable. The entry $\mathbf{D}[i, j]$ is set to 1 if instrumental variable j affects response variable i, and 0 otherwise. For identifiability, each response variable must have at least one instrumental variable that affects only that specific response (refer to Section (4)). In other words, for each row in **D**, there must be at least one column with a 1, and that column must contain 0's in all other rows. When using **Syy**, **Beta**, and **SigmaHat** as inputs, this condition must be strictly satisfied. If not, an error will be thrown, halting the algorithm. However, when using

X, **Y**, or **Syy**, **Syx**, and **Sxx** as inputs, a warning will be issued if the condition is violated, but the method will proceed and users are cautioned to interpret the results.

- **n**: **n** is a positive integer input representing the number of datapoints or observations in the dataset. This input is only required when summary level data is given as input.
- **nIter**: **nIter** is a positive integer input representing the number of MCMC (Markov Chain Monte Carlo) sampling iterations. The default value is set to $10,000$.
- **nBurnin**: **nBurnin** is a non-negative integer input representing the number of samples to be discarded during the burn-in phase of MCMC sampling. It's important that **nBurnin** is less than **nIter**. The default value is set to $2,000$.
- **Thin**: **Thin** is a positive integer input denoting the thinning factor applied to posterior samples. Thinning reduces the number of samples retained from the MCMC process for efficiency. Thin should not exceed (**nIter - nBurnin**). The default value is set to 1.
- **prior**: **prior** is a parameter representing the prior assumption on the graph structure. It offers two options: "Threshold" or "Spike and Slab". The default is set to "Spike and Slab".
- **aRho**: **aRho** is a positive scalar input representing the first parameter of a beta distribution in model (2.3) for Spike and Slab prior assumption on **A**. The default value is set to 0.5.
- **bRho**: **bRho** is a positive scalar input representing the second parameter of a beta distribution in model (2.3) for Spike and Slab prior assumption on **A**. The default value is set to 0.5.
- **nu1**: **nu1** is a positive scalar input representing the multiplication factor in the variance of the spike part in the spike and slab distribution of matrix **A** in model (2.3). The default value is set to 0.0001.
- **aPsi**: **aPsi** is a positive scalar input representing the first parameter of a beta distribution in model (2.3) for Spike and Slab prior assumption on **B**. The default value is set to 0.5.
- **bPsi**: **bPsi** is a positive scalar input representing the second parameter of a beta distribution in model (2.3) for Spike and Slab prior assumption on **B**. The default value is set to 0.5.
- **nu2**: **nu2** is a positive scalar input representing the multiplication factor in the variance of the spike part in the spike and slab distribution of matrix **B** in model (2.3). The default value is set to 0.0001.
- **aSigma**: **aSigma** is a positive scalar input corresponding to the first parameter of an inverse gamma distribution, which is associated to the variance of the model in model (2.2) & (2.3). The default value is set to 0.01.
- **bSigma**: **bSigma** is a positive scalar input corresponding to the second parameter of an inverse gamma distribution, which is associated to the variance of the model in model (2.2) & (2.3). The default value is set to 0.01.
- **PropVarA**: **PropVarA** is a positive scalar input corresponding to the variance of the normal distribution used for proposing terms within the **A** matrix by Metropolis-Hastings (M-H) algorithm. The default value is set to 0.01.

- **PropVarB**: **PropVarB** is a positive scalar input corresponding to the variance of the normal distribution used for proposing terms within the **B** matrix by Metropolis-Hastings (M-H) algorithm. The default value is set to 0.01.

Instructions to the Users:

1. **RGM** offers three data input options: individual-level data (**X** and **Y** matrices), summary-level data (**Syy**, **Syx**, **Sxx**) and a custom format (**Sxx**, **Beta**, **SigmaHat**). It prioritizes **X** and **Y** matrices, then **Syy**, **Syx**, **Sxx**, and finally, **Sxx**, **Beta**, **SigmaHat**. Users can select any of these data formats to suit their needs and don't have to specify all of them, allowing flexibility based on data availability. If none of these formats are provided, **RGM** prompts for valid data input.

2. Users must provide their values for **D** and **n**, two essential input parameters for **RGM**.

3. Users have the flexibility to customize the rest input parameters in **RGM**, allowing them to fine-tune the model to better fit their data and research objectives. While default values are available, users can experiment with these parameters to assess their impact on model performance.

3.1.2 Outputs

We will now specify outputs of **RGM** and will give brief descriptions about them.

- **AEst**: **AEst** is a matrix of dimensions $p \times p$, representing the estimated causal effects or strengths between the response variables. Here "p" signifies the number of response variable.

- **BEst**: **BEst** is a matrix of dimensions $p \times k$, representing the estimated causal effects or strengths between the response variables and the instrument variables. Each row corresponds to a specific response variable, and each column corresponds to a particular instrument variable. Here, "p" signifies the number of response variables and "k" represents the number of instrument variables.

- **zAEst**: **zAEst** is a binary adjacency matrix of dimensions $p \times p$, indicating the graph structure between the response variables. Each entry in the matrix represents the presence (1) or absence (0) of a causal link between the corresponding response variables. Here "p" signifies the number of response variable. This is obtained by thresholding **GammaEst** at 0.5.

- **zBEst**: **zBEst** is a binary adjacency matrix of dimensions $p \times k$, illustrating the graph structure between the response variables and the instrument variables. Here, "p" signifies the number of response variables and "k" represents the number of instrument variables. Each row corresponds to a specific response variable, and each column corresponds to a particular instrument variable. The presence of a causal link is denoted by 1, while the absence is denoted by 0. This is obtained by thresholding **PhiEst** at 0.5.

- **Graph**: A graph object that visually represents the estimated causal network by integrating **AEst** and **zAEst**. The graph consists of directed edges that indicate causal relationships between response variables. The edge weights correspond to effect sizes from **AEst**, while the structure of the graph is defined by **zAEst**, which already contains binary values (0 or 1) after thresholding at 0.5. This visualization provides an intuitive way to analyze both

the strength and structure of causal effects within the network. Users can plot this graph to explore the causal relationships effectively.

- **A0Est**: **A0Est** is a matrix of dimensions $p \times p$, representing the estimated causal effects or strengths between response variables before thresholding. Here "p" signifies the number of response variable. This output is particularly relevant for cases where the "Threshold" prior assumption is utilized.

- **B0Est**: **B0Est** is a matrix of dimensions $p \times k$, representing the estimated causal effects or strengths between the response variables and the instrument variables before thresholding. This output is particularly relevant for cases where the "Threshold" prior assumption is utilized. Here, "p" signifies the number of response variables and "k" represents the number of instrument variables. Each row corresponds to a specific response variable, and each column corresponds to a particular instrument variable.

- **GammaEst**: **GammaEst** is a matrix of dimensions $p \times p$, representing the estimated probabilities of edges between response variables in the graph structure. Here "p" signifies the number of response variable. Each entry in the matrix indicates the probability of a causal link between the corresponding response variables.

- **TauEst**: **TauEst** is a matrix of dimensions $p \times p$, representing the estimated variances of causal interactions between response variables. Here "p" signifies the number of response variable. Each entry in the matrix corresponds to the variance of the causal effect between the corresponding response variables.

- **PhiEst**: **PhiEst** is a matrix of dimensions $p \times k$, representing the estimated probabilities of edges between response and instrument variables in the graph structure. Here, "p" signifies the number of response variables and "k" represents the number of instrument variables. Each row corresponds to a specific response variable, and each column corresponds to a particular instrument variable.

- **EtaEst**: **EtaEst** is a matrix of dimensions $p \times k$, representing the estimated variances of causal interactions between response and instrument variables. Here, "p" signifies the number of response variables and "k" represents the number of instrument variables. Each row corresponds to a specific response variable, and each column corresponds to a particular instrument variable.

- **tAEst**: **tAEst** is a scalar value representing the estimated thresholding value of causal interactions between response variables. This output is relevant when using the "Threshold" prior assumption.

- **tBEst**: **tBEst** is a scalar value representing the estimated thresholding value of causal interactions between response and instrument variables. This output is applicable when using the "Threshold" prior assumption.

- **SigmaEst**: **SigmaEst** is a vector of length p, representing the estimated variances of each response variable. Here "p" signifies the number of response variables. Each element in the vector corresponds to the variance of a specific response variable.

- **AccptA**: **AccptA** is the percentage of accepted entries in the **A** matrix, which represents the causal interactions between response variables. This metric indicates the proportion of proposed changes that were accepted during the sampling process.

- **AccptB**: **AccptB** is the percentage of accepted entries in the **B** matrix, which represents the causal interactions between response and instrument variables. This metric indicates the proportion of proposed changes that were accepted during the sampling process.

- **AccpttA**: **AccpttA** is the percentage of accepted thresholding values for causal interactions between response variables when using the "Threshold" prior assumption. This metric indicates the proportion of proposed thresholding values that were accepted during the sampling process.

- **AccpttB**: **AccpttB** is the percentage of accepted thresholding values for causal interactions between response and instrument variables when using the "Threshold" prior assumption. This metric indicates the proportion of proposed thresholding values that were accepted during the sampling process.

- **LLPst**: **LLPst** is a vector containing the posterior log-likelihoods of the model. Each element in the vector represents the log-likelihood of the model given the observed data and the estimated parameters.

- **GammaPst**: **GammaPst** comprises the posterior samples of the causal network among the response variables. It has dimensions $p \times p \times n_{pst}$, where n_{pst} is the number of posterior samples and p denotes the number of response variables. Each slice of the array represents a $p \times p$ matrix indicating the estimated causal network among the response variables at a particular iteration. This is a vital input for the **NetworkMotif** function to quantify uncertainty for a specific network.

- **RhoEst**: **RhoEst** is a matrix of dimensions $p \times p$, representing the estimated Bernoulli success probabilities of causal interactions between response variables when using the "Spike and Slab" prior assumption. Here "p" signifies the number of response variable. Each entry in the matrix corresponds to the success probability of a causal interaction between the corresponding response variables.

- **PsiEst**: **PsiEst** is a matrix of dimensions $p \times k$, representing the estimated Bernoulli success probabilities of causal interactions between response and instrument variables when using the "Spike and Slab" prior assumption. Here, "p" signifies the number of response variables and "k" represents the number of instrument variables. Each row in the matrix corresponds to a specific response variable, and each column corresponds to a particular instrument variable.

3.2 NetworkMotif

3.2.1 Inputs

Next, we'll outline the inputs required for **NetworkMotif** and provide concise descriptions for each of them.

- **Gamma**: **Gamma** represents a $p \times p$ matrix that signifies a specific network motif (i.e., the adjacency matrix of a subgraph) among the response variables, where "p" represents the number of response variables. This matrix is the focus of uncertainty quantification.

- **GammaPst**: **GammaPst** comprises the posterior samples of the causal network among the response variables. It has dimensions $p \times p \times n_{pst}$, where n_{pst} is the number of posterior samples and p denotes the number of response variables. Each slice of the array represents a

$p \times p$ adjacency matrix indicating the estimated causal network among the response variables at a particular iteration. **GammaPst** may be obtained from the **RGM** function.

3.2.2 Output

The **NetworkMotif** function calculates the posterior probability for the provided network motif as a measure of uncertainty. A value close to 1 indicates that the given network motif is frequently observed in the posterior samples, while a value close to 0 suggests that the given network structure is rarely observed in the posterior samples.

4 Instrumental Variables in Mendelian Randomization

In Mendelian Randomization (MR) studies, the validity of instrumental variables (IVs) is fundamental for ensuring accurate causal inference. For an instrument to be considered valid, it must satisfy the following three key assumptions:

1. **Relevance**: The instrument, denoted as $\mathbf{Z}$, must be associated with the exposure variable $\mathbf{X}$. This condition ensures that $\mathbf{Z}$ has a significant effect on $\mathbf{X}$, mathematically expressed as:
$$\mathrm{Cov}(\mathbf{Z}, \mathbf{X}) \neq 0.$$
2. **Independence**: The instrument $\mathbf{Z}$ must be independent of any confounding variables $\mathbf{U}$ that influence both the exposure $\mathbf{X}$ and the outcome $\mathbf{Y}$. This ensures that $\mathbf{Z}$ is not related to any confounders that could introduce bias, which is expressed as:
$$\mathrm{Cov}(\mathbf{Z}, \mathbf{U}) = 0.$$
3. **Exclusion Restriction**: The instrument $\mathbf{Z}$ should not have a direct effect on the outcome $\mathbf{Y}$, except through the exposure $\mathbf{X}$. This implies that the effect of $\mathbf{Z}$ on $\mathbf{Y}$ operates solely through $\mathbf{X}$, ensuring no other pathways influence the outcome. Mathematically, this is expressed as:
$$\mathrm{Cov}(\mathbf{Z}, \mathbf{Y} \mid \mathbf{X}) = 0.$$

An instrument may have effects on multiple traits. However, we require each trait to have at least one valid instrument for model identifiability.

5 Implemenation of RGM and NetworkMotif

In this section, we consider illustrating some functionalities of the **MR.RGM** package, where we implement the primary functions **RGM** and **NetworkMotif** to some simulated examples.

5.1 Installation of the Package from CRAN

The following lines of `R` code shows the installation of the package from CRAN [1].

```
install.packages("MR.RGM")

# loading the package
library(MR.RGM)
```

5.2 Simulating Data for Implementation

We generate simulated data based on the assumed model and then proceed to demonstrate the implementation of **RGM** and **NetworkMotif**.

```
# Model: Y = AY + BX + E

# Set seed
set.seed(9154)

# Number of data points
n = 10000

# Number of response variables and number of instrument variables
p = 5
k = 6

# Initialize causal interaction matrix between response variables
A = matrix(sample(c(-0.1, 0.1), p^2, replace = TRUE), p, p)

# Diagonal entries of A matrix will always be 0
diag(A) = 0

# Make the network sparse
A[sample(which(A!=0), length(which(A!=0))/2)] = 0

# Create D matrix (Indicator matrix where each row corresponds to a response
   variable
# and each column corresponds to an instrument variable)
D = matrix(0, nrow = p, ncol = k)

# Manually assign values to D matrix
D[1, 1:2] = 1  # First response variable is influenced by the first 2 instruments
D[2, 3] = 1    # Second response variable is influenced by the 3rd instrument
D[3, 4] = 1    # Third response variable is influenced by the 4th instrument
D[4, 5] = 1    # Fourth response variable is influenced by the 5th instrument
D[5, 6] = 1    # Fifth response variable is influenced by the 6th instrument


# Initialize B matrix
B = matrix(0, p, k)  # Initialize B matrix with zeros

# Calculate B matrix based on D matrix
for (i in 1:p) {
   for (j in 1:k) {
     if (D[i, j] == 1) {
       B[i, j] = 1  # Set B[i, j] to 1 if D[i, j] is 1
     }
   }
 }



# Create variance-covariance matrix
Sigma = 1 * diag(p)

Mult_Mat = solve(diag(p) - A)
```

```
Variance = Mult_Mat %*% Sigma %*% t(Mult_Mat)

# Generate instrument data matrix
X = matrix(runif(n * k, 0, 5), nrow = n, ncol = k)

# Initialize response data matrix
Y = matrix(0, nrow = n, ncol = p)

# Generate response data matrix based on instrument data matrix
for (i in 1:n) {

 Y[i, ] = MASS::mvrnorm(n = 1, Mult_Mat %*% B %*% X[i, ], Variance)

}

# Print true causal interaction matrices between response variables and between
    response and instrument variables
A

#>      [,1] [,2] [,3] [,4] [,5]
#> [1,]  0.0 -0.1  0.0  0.0  0.1
#> [2,]  0.1  0.0 -0.1  0.1  0.1
#> [3,]  0.0 -0.1  0.0  0.0  0.1
#> [4,]  0.0 -0.1  0.0  0.0  0.0
#> [5,]  0.0  0.1  0.0  0.0  0.0

B

#>      [,1] [,2] [,3] [,4] [,5] [,6]
#> [1,]    1    1    0    0    0    0
#> [2,]    0    0    1    0    0    0
#> [3,]    0    0    0    1    0    0
#> [4,]    0    0    0    0    1    0
#> [5,]    0    0    0    0    0    1
```

5.3 Using RGM Function in MR.RGM

Initially, we demonstrate the application of the **RGM** function on the simulated dataset under varied prior assumptions, followed by a comparison of the graphical structures generated by **RGM** with the actual graph structures existing between response variables and between response and instrument variables.

```
# Apply RGM on individual level data with Threshold prior
Output1 = RGM(X = X, Y = Y, D = D, prior = "Threshold")

# Calculate summary level data
Syy = t(Y) %*% Y / n
Syx = t(Y) %*% X / n
Sxx = t(X) %*% X / n

# Apply RGM on summary level data for Spike and Slab Prior
Output2 = RGM(Syy = Syy, Syx = Syx, Sxx = Sxx, D = D, n = 10000, prior = "Spike
    and Slab")

# Calculate Beta and SigmaHat
# Centralize Data
Y = t(t(Y) - colMeans(Y))
```

```
X = t(t(X) - colMeans(X))

# Calculate Sxx
Sxx = t(X) %*% X / n

# Generate Beta matrix and SigmaHat
Beta = matrix(0, nrow = p, ncol = k)
SigmaHat = matrix(0, nrow = p, ncol = k)

for (i in 1:p) {

    for (j in 1:k) {

        fit = lm(Y[, i] ~ X[, j])

        Beta[i, j] =  fit$coefficients[2]

        SigmaHat[i, j] = sum(fit$residuals^2) / n

        }

 }


# Apply RGM on Sxx, Beta and SigmaHat for Spike and Slab Prior
Output3 = RGM(Sxx = Sxx, Beta = Beta, SigmaHat = SigmaHat,
           D = D, n = 10000, prior = "Spike and Slab")
```

The estimated causal interaction matrices between response variables are obtained as follows,

```
Output1$AEst
#>           [,1]        [,2]       [,3]      [,4]       [,5]
#> [1,] 0.0000000 -0.11032661  0.0000000 0.0000000 0.10676811
#> [2,] 0.0991208  0.00000000 -0.1104576 0.1002182 0.11012341
#> [3,] 0.0000000 -0.09370579  0.0000000 0.0000000 0.09747664
#> [4,] 0.0000000 -0.10185959  0.0000000 0.0000000 0.00000000
#> [5,] 0.0000000  0.10045256  0.0000000 0.0000000 0.00000000
Output2$AEst
#>              [,1]       [,2]          [,3]          [,4]        [,5]
#> [1,]  0.000000000 -0.1127249 -0.0006355133  0.0012237310 0.107520412
#> [2,]  0.099881480  0.0000000 -0.1079853541  0.0996589823 0.109275947
#> [3,] -0.001747246 -0.0929592  0.0000000000 -0.0006473297 0.099778267
#> [4,] -0.003863658 -0.1030056 -0.0024683725  0.0000000000 0.009191016
#> [5,]  0.001966818  0.1014136 -0.0055458038 -0.0050688662 0.000000000
Output3$AEst
#>             [,1]        [,2]         [,3]          [,4]       [,5]
#> [1,]  0.00000000 -0.08972628  0.039412442 -0.0006516754 0.09454632
#> [2,]  0.11040214  0.00000000 -0.112569739  0.0983975424 0.13676682
#> [3,]  0.01896308 -0.09780459  0.000000000  0.0100459671 0.11765744
#> [4,]  0.00000000 -0.11255315  0.001661851  0.0000000000 0.01358832
#> [5,] -0.00174675  0.12947084  0.011533919 -0.0025906139 0.00000000
```

The estimated causal network structures between the response variables are as follows,

```
Output1$zAEst
#>      [,1] [,2] [,3] [,4] [,5]
#> [1,]    0    1    0    0    1
#> [2,]    1    0    1    1    1
```

```
#> [3,]    0    1    0    0    1
#> [4,]    0    1    0    0    0
#> [5,]    0    1    0    0    0
Output2$zAEst
#>      [,1] [,2] [,3] [,4] [,5]
#> [1,]    0    1    0    0    1
#> [2,]    1    0    1    1    1
#> [3,]    0    1    0    0    1
#> [4,]    0    1    0    0    0
#> [5,]    0    1    0    0    0
Output3$zAEst
#>      [,1] [,2] [,3] [,4] [,5]
#> [1,]    0    1    0    0    1
#> [2,]    1    0    1    1    1
#> [3,]    0    1    0    0    1
#> [4,]    0    1    0    0    0
#> [5,]    0    1    0    0    0
```

We observe that the causal network structures inferred in the three outputs mentioned are identical. To gain a clearer understanding of the network, we compare the true network structure with the one estimated by **RGM**. Since the networks derived from all three outputs are consistent, we plot a single graph representing the estimated causal network.

```
# Define a function to create smaller arrowheads
smaller_arrowheads <- function(graph) {
  igraph::E(graph)$arrow.size = 0.60  # Adjust the arrow size value as needed
  return(graph)
}

# Create a layout for multiple plots
par(mfrow = c(1, 2))

# Plot the true causal network
plot(smaller_arrowheads(igraph::graph_from_adjacency_matrix((A != 0) * 1,
       mode = "directed")), layout = igraph::layout_in_circle,
          main = "True Causal Network")

# Plot the estimated causal network
plot(Output1$Graph, main = "Estimated Causal Network")
```

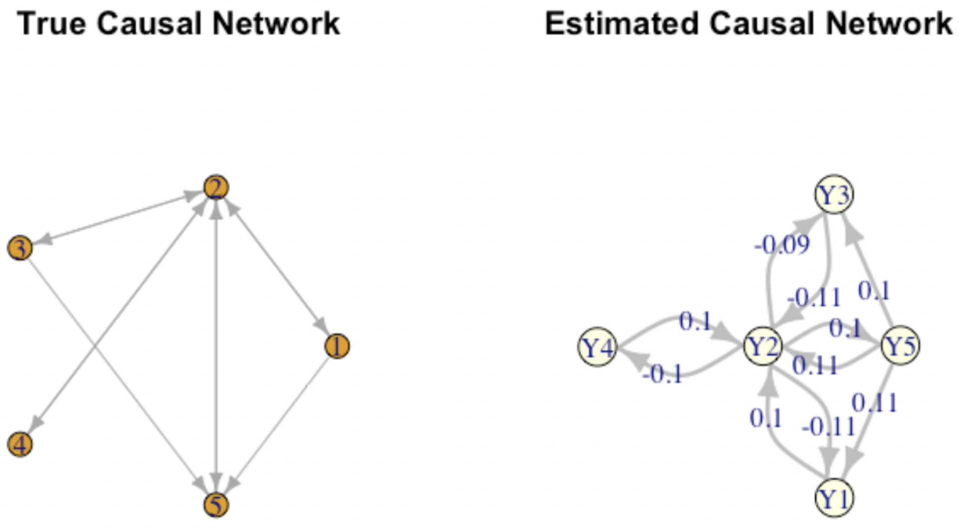

Figure 1: Comparison of the true causal network with the one generated by **RGM**.

We get the estimated causal interaction matrices between the response and the instrument variables from the outputs in the following way,

```
Output1$BEst
#>          [,1]     [,2]     [,3]      [,4]      [,5]      [,6]
#> [1,] 0.9935119 1.008009 0.000000 0.0000000 0.0000000 0.0000000
#> [2,] 0.0000000 0.000000 0.997496 0.0000000 0.0000000 0.0000000
#> [3,] 0.0000000 0.000000 0.000000 0.9998662 0.0000000 0.0000000
#> [4,] 0.0000000 0.000000 0.000000 0.0000000 0.9995511 0.0000000
#> [5,] 0.0000000 0.000000 0.000000 0.0000000 0.0000000 0.9982094
Output2$BEst
#>          [,1]   [,2]      [,3]      [,4]      [,5]     [,6]
#> [1,] 0.9937211 1.0075 0.0000000 0.0000000 0.0000000 0.000000
#> [2,] 0.0000000 0.0000 0.9964755 0.0000000 0.0000000 0.000000
#> [3,] 0.0000000 0.0000 0.0000000 0.9990566 0.0000000 0.000000
#> [4,] 0.0000000 0.0000 0.0000000 0.0000000 0.9987975 0.000000
#> [5,] 0.0000000 0.0000 0.0000000 0.0000000 0.0000000 1.002271
Output3$BEst
#>          [,1]     [,2]      [,3]      [,4]      [,5]      [,6]
#> [1,] 0.9902686 1.004035 0.0000000 0.0000000 0.0000000 0.0000000
#> [2,] 0.0000000 0.000000 0.9928588 0.0000000 0.0000000 0.0000000
#> [3,] 0.0000000 0.000000 0.0000000 0.9988565 0.0000000 0.0000000
#> [4,] 0.0000000 0.000000 0.0000000 0.0000000 0.9970532 0.0000000
#> [5,] 0.0000000 0.000000 0.0000000 0.0000000 0.0000000 0.9965687
```

The estimated graph structures between the response and the instrument variables are obtained as follows,

```
Output1$zBEst
#>      [,1] [,2] [,3] [,4] [,5] [,6]
#> [1,]    1    1    0    0    0    0
#> [2,]    0    0    1    0    0    0
#> [3,]    0    0    0    1    0    0
#> [4,]    0    0    0    0    1    0
#> [5,]    0    0    0    0    0    1
```

```
Output2$zBEst
#>      [,1] [,2] [,3] [,4] [,5] [,6]
#> [1,]    1    1    0    0    0    0
#> [2,]    0    0    1    0    0    0
#> [3,]    0    0    0    1    0    0
#> [4,]    0    0    0    0    1    0
#> [5,]    0    0    0    0    0    1
Output3$zBEst
#>      [,1] [,2] [,3] [,4] [,5] [,6]
#> [1,]    1    1    0    0    0    0
#> [2,]    0    0    1    0    0    0
#> [3,]    0    0    0    1    0    0
#> [4,]    0    0    0    0    1    0
#> [5,]    0    0    0    0    0    1
```

We can plot the log-likelihoods from the outputs in the following way,

```
plot(Output1$LLPst, type = 'l', xlab = "Iterations", ylab = "Log-likelihood")
```

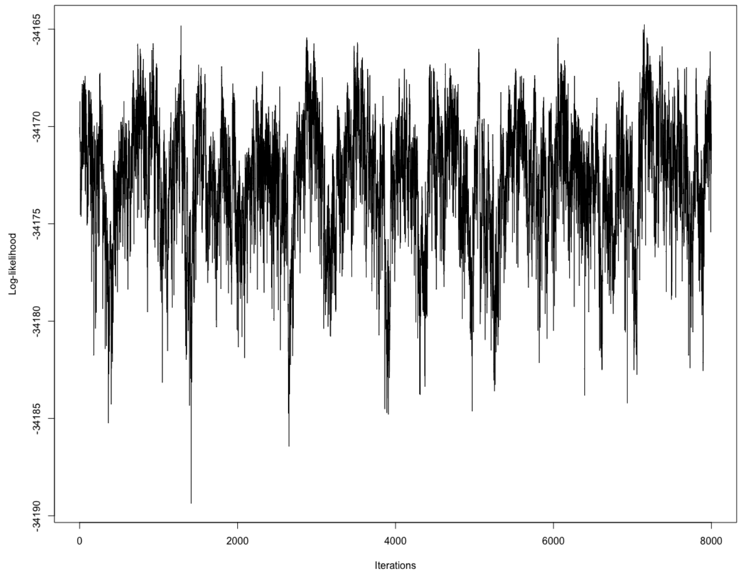

Figure 2: Posterior samples of log-likelihood vs Iterations.

```
plot(Output2$LLPst, type = 'l', xlab = "Iterations", ylab = "Log-likelihood")
```

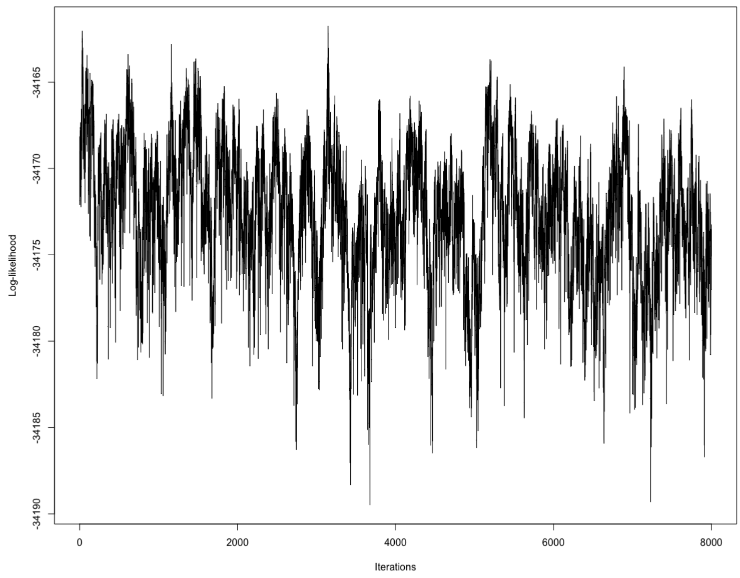

Figure 3: Posterior samples of log-likelihood vs Iterations.

```
plot(Output3$LLPst, type = 'l', xlab = "Iterations", ylab = "Log-likelihood")
```

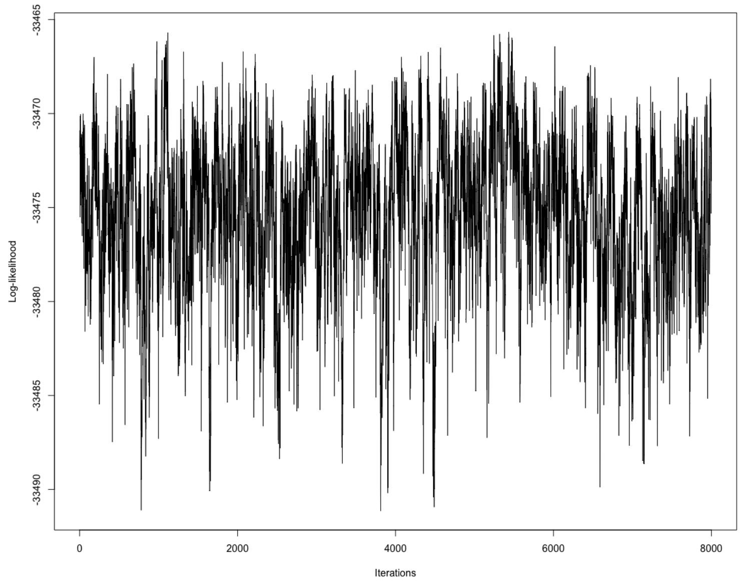

Figure 4: Posterior samples of log-likelihood vs Iterations.

5.4 Expanded Simulation Setup

In real-world scenarios, it is common to encounter a large number of instrumental variables (IVs), each explaining only a small proportion of the trait variance. To assess our method in such scenarios, we have expanded our earlier simulations with the following additional elements:

5.4.1 Initial Setup with Numerous IVs

We start with 100 IVs for each response variable, each IV having a small effect on the trait. This setup mimics the situation where each IV contributes minimally to the overall trait variance, as often observed in real GWAS studies.

5.4.2 Dimensionality Reduction

To manage the high dimensionality of the data and focus on the most significant aspects of the variability, we perform PCA on each set of SNPs associated with a response variable. We select the top 20 principal components (PCs) from each PCA. This step reduces the complexity of the data while retaining the key features that explain the majority of the variance. By combining the top PCs from each response variable, we form a condensed matrix `compact_X`. This matrix aggregates the instrumental variables into a more manageable form, facilitating a more efficient analysis.

5.4.3 Revised Summary Level Data

Using `compact_X`, we calculate new summary level data (`Sxx_compact` and `Syx_compact`) for the RGM function application.

5.4.4 Updated R Code

Here is the updated R code reflecting these changes:

```
# Load necessary libraries
library(MASS)
library(igraph)

# Set seed for reproducibility
set.seed(9154)

# Number of data points
n = 10000

# Number of response variables
p = 5

# Number of SNPs per response variable
num_snps_per_y = 100

# Total number of SNPs
k = num_snps_per_y * p

# Initialize causal interaction matrix between response variables
A = matrix(sample(c(-0.1, 0.1), p^2, replace = TRUE), p, p)
diag(A) = 0
A[sample(which(A != 0), length(which(A != 0)) / 2)] = 0

# Create D matrix (Indicator matrix where each row corresponds to a response
   variable
# and each column corresponds to an instrument variable)
D = matrix(0, nrow = p, ncol = k)

# Assign values to D matrix using a loop
for (run in 1:p) {
```

```
  D[run, ((run - 1) * 100 + 1):(run * 100)] = 1
}

# Initialize B matrix
B = matrix(0, p, k)  # Initialize B matrix with zeros

# Calculate B matrix based on D matrix
for (i in 1:p) {
  for (j in 1:k) {
    if (D[i, j] == 1) {
      B[i, j] = 1  # Set B[i, j] to 1 if D[i, j] is 1
    }
  }
}

# Calculate Variance-Covariance matrix
Sigma = diag(p)
Mult_Mat = solve(diag(p) - A)
Variance = Mult_Mat %*% Sigma %*% t(Mult_Mat)

# Generate instrument data matrix (X)
X = matrix(rnorm(n * k, 0, 1), nrow = n, ncol = k)

# Initialize response data matrix (Y)
Y = matrix(0, nrow = n, ncol = p)

# Generate response data matrix based on instrument data matrix
for (i in 1:n) {
  Y[i, ] = MASS::mvrnorm(n = 1, Mult_Mat %*% B %*% X[i, ], Variance)
}

# Calculate summary level data
Syy = t(Y) %*% Y / n
Syx = t(Y) %*% X / n
Sxx = t(X) %*% X / n

# Perform PCA for each response variable to get top 20 PCs
top_snps_list = list()
for (i in 1:p) {
  X_sub = X[, (num_snps_per_y * (i - 1) + 1):(num_snps_per_y * i)]
  pca = prcomp(X_sub, center = TRUE, scale. = TRUE)
  top_20_pcs = pca$x[, 1:20]
  top_snps_list[[i]] = top_20_pcs
}

# Combine the top PCs from all response variables
compact_X = do.call(cbind, top_snps_list)

# Calculate summary level data based on compact_X
Sxx_compact = t(compact_X) %*% compact_X / n
Syx_compact = t(Y) %*% compact_X / n

# Create D_New
D_New = matrix(0, nrow = p, ncol = 20 * p)

# Assign values to D matrix using a loop
for (run in 1:p) {
  D_New[run, ((run - 1) * 20 + 1):(run * 20)] = 1
}
```

```
# Apply RGM on summary level data for Spike and Slab Prior using the compact_X
    matrix
Output = RGM(Syy = Syy, Syx = Syx_compact, Sxx = Sxx_compact, D = D_New, n = n,
    prior = "Spike and Slab")

# Print estimated causal interaction matrices
Output$AEst
#>               [,1]        [,2]         [,3]         [,4]       [,5]
#> [1,]  0.0000000000 -0.10304239  0.012795897  0.006911765 0.09119095
#> [2,]  0.1102430697  0.00000000 -0.101718794  0.095093965 0.10762267
#> [3,] -0.0039579652 -0.10801784  0.000000000 -0.008388722 0.10255348
#> [4,] -0.0013919956 -0.09076408  0.011536478  0.000000000 0.01075220
#> [5,] -0.0005533104  0.10848253 -0.002160112 -0.011725145 0.00000000
Output$zAEst
#>      [,1] [,2] [,3] [,4] [,5]
#> [1,]    0    1    0    0    1
#> [2,]    1    0    1    1    1
#> [3,]    0    1    0    0    1
#> [4,]    0    1    0    0    0
#> [5,]    0    1    0    0    0

# Create a layout for multiple plots
par(mfrow = c(1, 2))

# Plot the true causal network
plot(smaller_arrowheads(igraph::graph_from_adjacency_matrix((A != 0) * 1, mode = "
    directed")), layout = igraph::layout_in_circle, main = "True Causal Network")

# Plot the estimated causal network
plot(Output$Graph, main = "Estimated Causal Network")
```

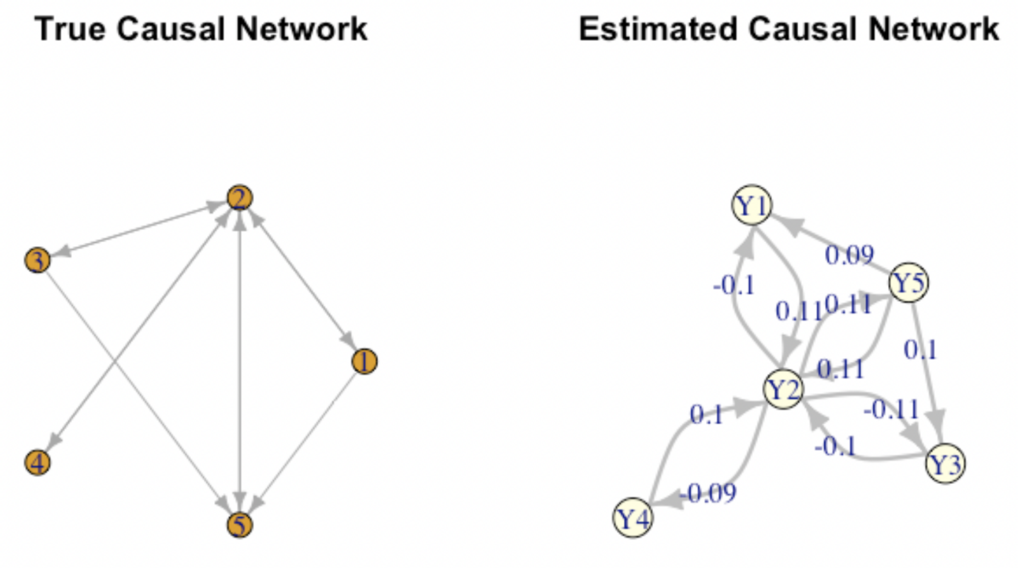

Figure 5: Comparison of the true causal network with the one generated by **RGM**.

Our approach again yields very promising results, which demonstrates that our method could be useful in complex scenarios with many weak IVs.

The dimension reduction technique (PCA) proves to be effective. This approach can be broadly applied to similar problems where dimensionality reduction is necessary. By leveraging PCA or other dimension reduction methods, researchers can efficiently manage large sets of IVs and apply our algorithm to gain valuable insights into causal relationships.

5.5 Using NetworkMotif Function in MR.RGM

Next, we present the implementation of the **NetworkMotif** function. We begin by defining a random subgraph among the response variables. Subsequently, we collect **GammaPst** arrays from various outputs and proceed to execute **NetworkMotif** based on these arrays.

```
# Start with a random subgraph
Gamma = matrix(0, nrow = p, ncol = p)
Gamma[5, 2] = Gamma[3, 5] = Gamma[2, 3] = 1

# Plot the subgraph to get an idea about the causal network
plot(smaller_arrowheads(igraph::graph_from_adjacency_matrix(Gamma,
       mode = "directed")), layout = igraph::layout_in_circle,
          main = "Subgraph")
```

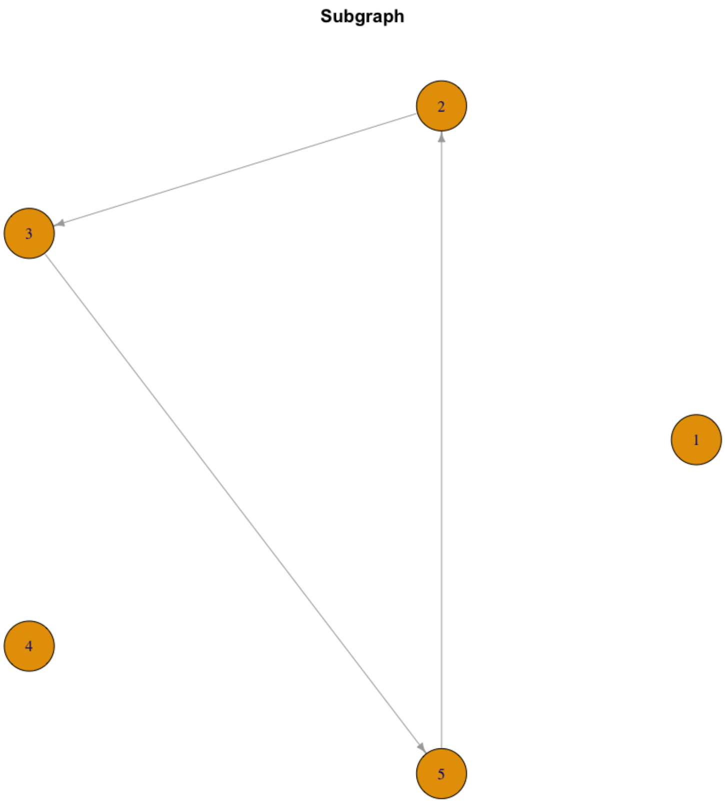

Figure 6: Subgraph between the response variables for which we want to caluclate posterior probability for.

```
# Store the GammaPst arrays from outputs
GammaPst1 = Output1$GammaPst
GammaPst2 = Output2$GammaPst
GammaPst3 = Output3$GammaPst

# Get the posterior probabilities of Gamma with these GammaPst matrices
NetworkMotif(Gamma = Gamma, GammaPst = GammaPst1)
#> [1] 1
NetworkMotif(Gamma = Gamma, GammaPst = GammaPst2)
#> [1] 0.3795
NetworkMotif(Gamma = Gamma, GammaPst = GammaPst3)
#> [1] 0.485125
```

6 Computational complexity reduction for RGM

6.1 Calculation of $\det(\mathbf{I_p} - \mathbf{A})$ and $(\mathbf{I_p} - \mathbf{A})^{-1}$

For MCMC sampling we have to calculate $\det(\mathbf{I_p} - \mathbf{A})$ while sampling each entry of $\mathbf{A}$. So, we have to calculate the determinant $p \times (p-1)$ many times inside each iteration and if p is large then this is heavily time consuming. In order to reduce complexity we have implemented woodbury matrix determinant lemma to calculate $\det(\mathbf{I_p} - \mathbf{A})$. Woodbury matrix determinant lemma says:

$$\det(\mathbf{A} + \mathbf{u}\mathbf{v}^T) = (1 + \mathbf{v}^T\mathbf{A}^{-1}\mathbf{u})\det(\mathbf{A}) \tag{6.1}$$

Now suppose we want to sample (i, j)th entry of $\mathbf{A}$. Suppose, we get $\mathbf{A}^*$ by replacing a_{ij} ((i, j)th entry of $\mathbf{A}$) with a^*_{ij} and keeping all other entries same as $\mathbf{A}$. Now, we want to calculate $\det(\mathbf{I_p} - \mathbf{A}^*)$. Now instead of directly calculating this, we will try to calculate this from $\det(\mathbf{I_p} - \mathbf{A})$ and in this way we will keep on updating this. Thus, we have:

$$\det(\mathbf{I_p} - \mathbf{A}^*) = \det(\mathbf{I_p} - \mathbf{A} + \mathbf{A} - \mathbf{A}^*) \tag{6.2}$$

Now the (i, j)th entry of $(\mathbf{A} - \mathbf{A}^*)$ is $(a_{ij} - a^*_{ij})$ and the rest are 0. Thus, $(\mathbf{A} - \mathbf{A}^*)$ can be written as $\mathbf{u}\mathbf{v}^T$, where $\mathbf{u}$ is a column vector whose ith entry is $(a_{ij} - a^*_{ij})$ and the rest are 0 and $\mathbf{v}$ is a column vector whose jth entry is 1 and the rest are 0. Thus, from equation (6.2), we have:

$$\begin{aligned}\det(\mathbf{I_p} - \mathbf{A}^*) &= \det(\mathbf{I_p} - \mathbf{A} + \mathbf{A} - \mathbf{A}^*) \\ &= \det(\mathbf{I_p} - \mathbf{A} + \mathbf{u}\mathbf{v}^T) \\ &= (1 + \mathbf{v}^T(\mathbf{I_p} - \mathbf{A})^{-1}\mathbf{u}).\det(\mathbf{I_p} - \mathbf{A}) \quad \text{[Using equation (6.1)]} \\ &= (1 + (\mathbf{I_p} - \mathbf{A})^{-1}_{(j,i)} \times (a_{ij} - a^*_{ij})).\det(\mathbf{I_p} - \mathbf{A}) \end{aligned} \tag{6.3}$$

where, $(\mathbf{I_p} - \mathbf{A})^{-1}_{(j,i)}$ denotes the (j, i)th entry of $(\mathbf{I_p} - \mathbf{A})^{-1}$. Now, we want to calculate $(\mathbf{I_p} - \mathbf{A}^*)^{-1}$, but instead of calculating it directly we will calculate it from $(\mathbf{I_p} - \mathbf{A})^{-1}$. We will use woodbury matrix identity which states:

$$(\mathbf{A} + \mathbf{UCV})^{-1} = \mathbf{A}^{-1} - \mathbf{A}^{-1}\mathbf{U}(\mathbf{C}^{-1} + \mathbf{V}\mathbf{A}^{-1}\mathbf{U})^{-1}\mathbf{V}\mathbf{A}^{-1} \tag{6.4}$$

Thus, we can calculate $(\mathbf{I_p} - \mathbf{A}^*)^{-1}$ in the following way:

$$\begin{aligned}(\mathbf{I_p} - \mathbf{A}^*)^{-1} &= (\mathbf{I_p} - \mathbf{A} + \mathbf{A} - \mathbf{A}^*)^{-1} \\ &= (\mathbf{I_p} - \mathbf{A} + \mathbf{u}\mathbf{v}^\mathbf{T})^{-1} \\ &= (\mathbf{I_p} - \mathbf{A})^{-1} - (\mathbf{I_p} - \mathbf{A})^{-1}\mathbf{u}(1 + \mathbf{v}^T(\mathbf{I_p} - \mathbf{A})^{-1}\mathbf{u})^{-1}\mathbf{v}^T(\mathbf{I_p} - \mathbf{A})^{-1} \text{ [Using equation (6.4)]} \\ &= (\mathbf{I_p} - \mathbf{A})^{-1} - \frac{a_{ij} - a^*_{ij}}{1 + (a_{ij} - a^*_{ij}).(\mathbf{I_p} - \mathbf{A})^{-1}_{(j,i)}}.(\mathbf{I_p} - \mathbf{A})^{-1}_{(.,i)} \times (\mathbf{I_p} - \mathbf{A})^{-1}_{(j,.)} \qquad (6.5)\end{aligned}$$

where, $(\mathbf{I_p} - \mathbf{A})^{-1}_{(.,i)}$ indicates ith coumn of $(\mathbf{I_p} - \mathbf{A})^{-1}$, $(\mathbf{I_p} - \mathbf{A})^{-1}_{(j,.)}$ indicates jth row of $(\mathbf{I_p} - \mathbf{A})^{-1}$ and $(\mathbf{I_p} - \mathbf{A})^{-1}_{(j,i)}$ indicates (j, i)th entry of $(\mathbf{I_p} - \mathbf{A})^{-1}$. So, we have implemented equation (6.3) to calculate $\det(\mathbf{I_p} - \mathbf{A}^*)$ from $\det(\mathbf{I_p} - \mathbf{A})$ and implemented equation (6.5) to calculate $(\mathbf{I_p} - \mathbf{A}^*)^{-1}$ from $(\mathbf{I_p} - \mathbf{A})^{-1}$ and we keep on updating them instead of directly calculating them everytime.

6.2 Reduction in complexity for matrix multiplication

For MCMC sampling we have to do some matrix multiplications and take trace while sampling each entry of $\mathbf{A}$. The trace terms which can be found in Section (9), are as follows:

$$\begin{aligned}\text{Trace1} &= -n \times \text{trace}(\mathbf{Syy} \times \mathbf{A^T} \times \mathbf{\Sigma^{-1}}) \\ \text{Trace2} &= -n \times \text{trace}(\mathbf{Syy} \times \mathbf{\Sigma^{-1}} \times \mathbf{A}) \\ \text{Trace3} &= n \times \text{trace}(\mathbf{Syy} \times \mathbf{A^T} \times \mathbf{\Sigma^{-1}} \times \mathbf{A}) \\ \text{Trace4} &= 2n \times \text{trace}(\mathbf{Syx} \times \mathbf{B^T} \times \mathbf{\Sigma^{-1}} \times \mathbf{A})\end{aligned}$$

where, $\mathbf{\Sigma} = \text{diag}(\sigma_1, \cdots, \sigma_p)$ and hence $\mathbf{\Sigma}^{-1} = \text{diag}(1/\sigma_1, \cdots, 1/\sigma_p)$. Now, suppose we want to sample (i, j)th entry of $\mathbf{A}$. We get $\mathbf{A}^*$ by replacing a_{ij} ((i, j)th entry of $\mathbf{A}$) with a^*_{ij} and keeping all other entries same as $\mathbf{A}$. Instead of calculating the trace values directly for $\mathbf{A}^*$, we will update it based on previous trace values in the following way:

$$\begin{aligned}\text{Trace1\_New} &= \text{Trace1} - n \times (a^*_{ij} - a_{ij}) \times \mathbf{\Sigma}^{-1}_{(i,i)} \times \mathbf{Syy}_{(i,j)} \\ \text{Trace2\_New} &= \text{Trace2} - n \times (a^*_{ij} - a_{ij}) \times \mathbf{\Sigma}^{-1}_{(i,i)} \times \mathbf{Syy}_{(j,i)} \\ \text{Trace3\_New} &= \text{Trace3} + n \times (a^*_{ij} - a_{ij}) \times \mathbf{\Sigma}^{-1}_{(i,i)} \times \Big(\mathbf{A}_{(i,.)} \times \mathbf{Syy}_{(.,j)} + \mathbf{Syy}_{(j,.)} \times \mathbf{A}^{*T}_{(i,.)}\Big) \\ \text{Trace4\_New} &= \text{Trace4} + 2n \times (a^*_{ij} - a_{ij}) \times \mathbf{\Sigma}^{-1}_{(i,i)} \times \Big(\mathbf{B}_{(i,.)} \times \mathbf{Syx}^T_{(j,.)}\Big)\end{aligned}$$

where, for any matrix $\mathbf{A}$, $\mathbf{A}_{(i,i)}$ denotes (i, i)th element of $\mathbf{A}$, $\mathbf{A}_{(i,.)}$ denotes ith row of $\mathbf{A}$ and $\mathbf{A}_{(.,i)}$ denotes ith column of $\mathbf{A}$. Similarly, while sampling each entry of $\mathbf{B}$, we have to calculate the following trace values:

$$\begin{aligned}\text{Trace5} &= -2n \times \text{trace}(\mathbf{Syx} \times \mathbf{B^T} \times \mathbf{\Sigma^{-1}} \times (\mathbf{I_p} - \mathbf{A})) \\ \text{Trace6} &= n \times \text{trace}(\mathbf{Sxx} \times \mathbf{B^T} \times \mathbf{\Sigma^{-1}} \times \mathbf{B})\end{aligned}$$

Now, suppose we want to sample (i,j)th entry of $\mathbf{B}$. We get $\mathbf{B}^*$ by replacing b_{ij} ((i,j)th entry of $\mathbf{B}$) with b^*_{ij} and keeping all other entries same as $\mathbf{B}$. Instead of calculating the trace values directly for $\mathbf{B}^*$, we will update it based on previous trace values in the following way:

$$\text{Trace5\_New} = \text{Trace5} - 2n \times (b^*_{ij} - b_{ij}) \times \mathbf{\Sigma}^{-1}_{(i,i)} \times ((\mathbf{I_p} - \mathbf{A})_{(i,.)} \times \mathbf{Syx}_{(.,j)})$$
$$\text{Trace6\_New} = \text{Trace6} + n \times (b^*_{ij} - b_{ij}) \times \mathbf{\Sigma}^{-1}_{(i,i)} \times (\mathbf{B}_{(i,.)} \times \mathbf{Sxx}_{(.,j)} + \mathbf{Sxx}_{(j,.)} \times \mathbf{B^*}^T_{(i,.)})$$

7 Computational Scalability of MR.RGM

To evaluate the computational scalability of **MR.RGM**, we conducted a series of experiments to assess its performance with varying numbers of traits and instrumental variables (IVs). We considered four scenarios:

1. **Fixing the number of traits at 5 and varying the number of IVs per trait:** We tested with 1, 5, 10, 25, 50, and 100 IVs per trait. The results are illustrated in Figure (7).

2. **Fixing the number of traits at 10 and varying the number of IVs per trait:** We used the same range of IVs as in the previous case. The corresponding results are shown in Figure (8).

3. **Fixing the number of IVs per trait at 5 and varying the number of traits:** We tested with 5, 10, 20, 50, and 75 traits. Results for this setup are presented in Figure (9).

4. **Fixing the number of IVs per trait at 10 and varying the number of traits:** We used the same range of traits as in the previous case. These results are depicted in Figure (10).

In each setup, the method was run 100 times, and for each run, **MR.RGM** performed 10,000 MCMC iterations with "Spike and Slab" prior. The median runtime was recorded. All experiments were conducted on an Apple M2 Pro processor with 16 GB of memory. **MR.RGM** demonstrated great computational efficiency compared to traditional Bayesian methods. **MR.RGM** scales relatively well with respect to the number of traits and IVs, as shown in the figures. This efficiency makes **MR.RGM** a practical choice for complex analyses involving numerous traits and instruments.

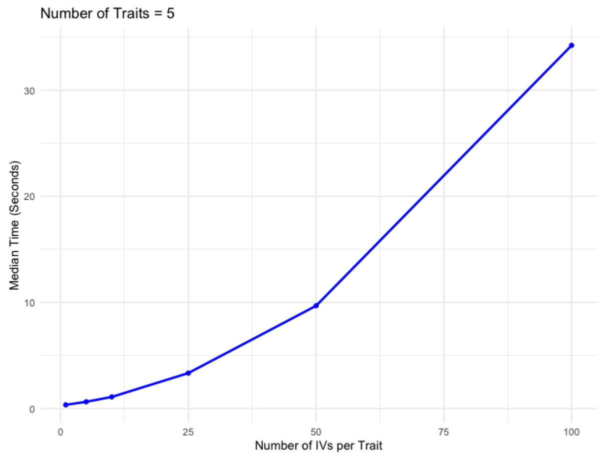

Figure 7: Median runtime for varying numbers of IVs per trait while fixing the number of traits at 5.

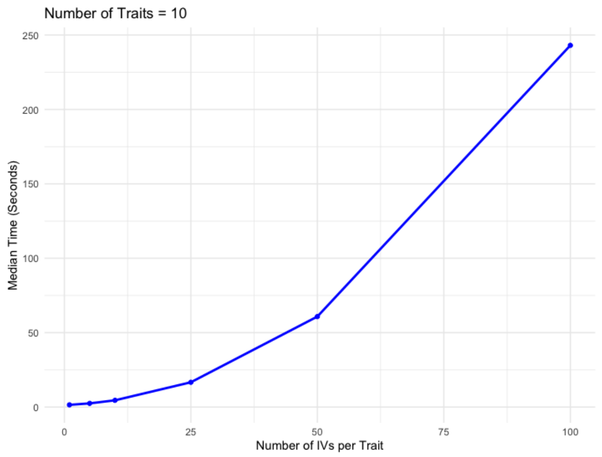

Figure 8: Median runtime for varying numbers of IVs per trait while fixing the number of traits at 10.

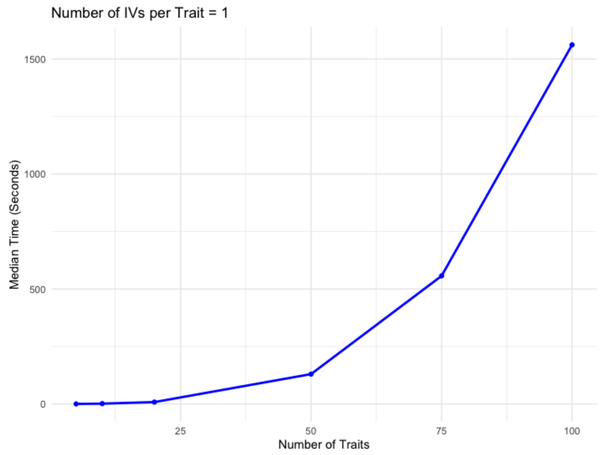

Figure 9: Median runtime for varying numbers of traits while fixing the number of IVs per trait at 1.

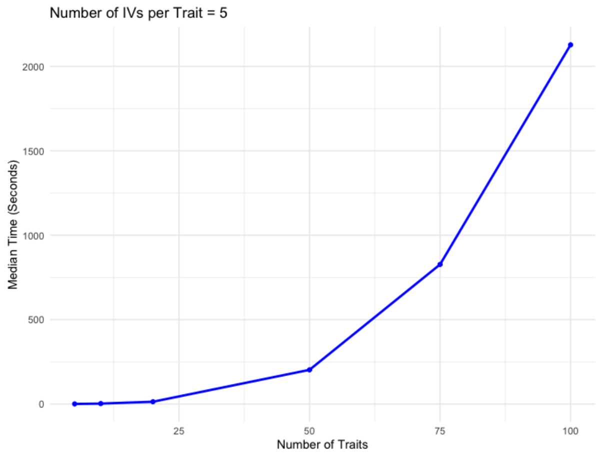

Figure 10: Median runtime for varying numbers of traits while fixing the number of IVs per trait at 5.

8 Sensitivity to the Assumption of Error Distributions

To evaluate the sensitivity of **MR.RGM** to different error distributions, we conducted a comparative analysis with several error types:

- **Normal Distribution:** Errors generated from normal distribution.
- **t-Distribution (df = 3):** Errors generated from a t-distribution with 3 degrees of freedom.
- **t-Distribution (df = 5):** Errors generated from a t-distribution with 5 degrees of freedom.
- **t-Distribution (df = 10):** Errors generated from a t-distribution with 10 degrees of freedom.

- **Laplace Distribution:** Errors generated from a Laplace distribution.

For each error distribution, **MR.RGM** was applied to the datasets, and the following metrics were computed:

- **True Positive Rate (TPR):** The proportion of true edges correctly identified by **MR.RGM** in the estimated network structure.
- **False Positive Rate (FPR):** The proportion of false edges incorrectly identified in the estimated network structure.
- **Mean Absolute Deviation (MAD):** The average deviation between the estimated causal effects and the true causal effects.

These metrics were calculated based on the estimated network structure and causal effects, with the process repeated $1,000$ times for each distribution.

8.1 Results

Table (1) summarizes the average TPR, FPR, and MAD for each error distribution. The results indicate that **MR.RGM** is not sensitive to the assumption of normal errors, as performance metrics are consistent across different error distributions. This suggests that **MR.RGM** performs robustly even when the error distribution deviates from normality.

Table 1: Performance of **MR.RGM** under different error distributions.

Error Distribution	TPR	FPR	MAD
Normal Distribution	0.86	0.0133	0.0169
t-Distribution (df = 3)	0.86	0.0200	0.0153
t-Distribution (df = 5)	0.92	0.0400	0.0154
t-Distribution (df = 10)	0.86	0.0467	0.0179
Laplace Distribution	0.87	0.0333	0.0164

9 Likelihood of the model based on summary level data

Now, we will illustrate the likelihood calculation when summary-level data is provided. The likelihood of the model, based on individual-level data, is expressed as follows:

$$
\begin{aligned}
&p\left(\{\mathbf{Y_i}\}_{i=1}^n|\{\mathbf{X_i}\}_{i=1}^n,\mathbf{A},\mathbf{B},\mathbf{\Sigma}\right)\\
=&\prod_{i=1}^n N(\mathbf{Y_i}|(\mathbf{I_p}-\mathbf{A})^{-1}\mathbf{B}\mathbf{X_i},(\mathbf{I_p}-\mathbf{A})^{-1}\mathbf{\Sigma}(\mathbf{I_p}-\mathbf{A})^{-\mathbf{T}})\\
=&\prod_{i=1}^n N((\mathbf{I_p}-\mathbf{A})\mathbf{Y_i}-\mathbf{B}\mathbf{X_i}|\mathbf{0},\mathbf{\Sigma}).|\det(\mathbf{I_p}-\mathbf{A})|\\
=&\prod_{i=1}^n(2\pi)^{-p/2}\det(\mathbf{\Sigma})^{-1/2}|\det(\mathbf{I_p}-\mathbf{A})|\exp(-\frac{1}{2}[(\mathbf{I_p}-\mathbf{A})\mathbf{Y_i}-\mathbf{B}\mathbf{X_i}]^\mathbf{T}\mathbf{\Sigma}^{-1}[(\mathbf{I_p}-\mathbf{A})\mathbf{Y_i}-\mathbf{B}\mathbf{X_i}])\\
=&(2\pi)^{-\frac{np}{2}}.\det(\mathbf{\Sigma})^{-\frac{n}{2}}.|\det(\mathbf{I_p}-\mathbf{A})|^\mathbf{n}.\exp(-\frac{1}{2}\sum_{i=1}^n[(\mathbf{I_p}-\mathbf{A})\mathbf{Y_i}-\mathbf{B}\mathbf{X_i}]^\mathbf{T}\mathbf{\Sigma}^{-1}[(\mathbf{I_p}-\mathbf{A})\mathbf{Y_i}-\mathbf{B}\mathbf{X_i}])\\
=&(2\pi)^{-\frac{np}{2}}.\det(\mathbf{\Sigma})^{-\frac{n}{2}}.|\det(\mathbf{I_p}-\mathbf{A})|^\mathbf{n}.\exp(-\frac{1}{2}\sum_{i=1}^n[\mathbf{Y_i^T}(\mathbf{I_p}-\mathbf{A})^\mathbf{T}\mathbf{\Sigma}^{-1}(\mathbf{I_p}-\mathbf{A})\mathbf{Y_i}\\
&\qquad-2\mathbf{X_i^T}\mathbf{B^T}\mathbf{\Sigma}^{-1}(\mathbf{I_p}-\mathbf{A})\mathbf{Y_i}+\mathbf{X_i^T}\mathbf{B^T}\mathbf{\Sigma}^{-1}\mathbf{B}\mathbf{X_i}])\\
=&(2\pi)^{-\frac{np}{2}}.\det(\mathbf{\Sigma})^{-\frac{n}{2}}.|\det(\mathbf{I_p}-\mathbf{A})|^\mathbf{n}.\exp(-\frac{1}{2}\sum_{i=1}^n[\mathrm{tr}(\mathbf{Y_i^T}(\mathbf{I_p}-\mathbf{A})^\mathbf{T}\mathbf{\Sigma}^{-1}(\mathbf{I_p}-\mathbf{A})\mathbf{Y_i})\\
&\qquad-2\mathrm{tr}(\mathbf{X_i^T}\mathbf{B^T}\mathbf{\Sigma}^{-1}(\mathbf{I_p}-\mathbf{A})\mathbf{Y_i})+\mathrm{tr}(\mathbf{X_i^T}\mathbf{B^T}\mathbf{\Sigma}^{-1}\mathbf{B}\mathbf{X_i})])\\
=&(2\pi)^{-\frac{np}{2}}.\det(\mathbf{\Sigma})^{-\frac{n}{2}}.|\det(\mathbf{I_p}-\mathbf{A})|^\mathbf{n}.\exp(-\frac{1}{2}\sum_{i=1}^n[\mathrm{tr}(\mathbf{Y_i}\mathbf{Y_i^T}(\mathbf{I_p}-\mathbf{A})^\mathbf{T}\mathbf{\Sigma}^{-1}(\mathbf{I_p}-\mathbf{A}))\\
&\qquad-2\mathrm{tr}(\mathbf{Y_i}\mathbf{X_i^T}\mathbf{B^T}\mathbf{\Sigma}^{-1}(\mathbf{I_p}-\mathbf{A}))+\mathrm{tr}(\mathbf{X_i}\mathbf{X_i^T}\mathbf{B^T}\mathbf{\Sigma}^{-1}\mathbf{B})])
\end{aligned}
$$

$$
\begin{aligned}
=&(2\pi)^{-\frac{np}{2}}.\det(\mathbf{\Sigma})^{-\frac{n}{2}}.|\det(\mathbf{I_p}-\mathbf{A})|^\mathbf{n}.\exp(-\frac{1}{2}[\mathrm{tr}(\sum_{i=1}^n\mathbf{Y_i}\mathbf{Y_i^T}(\mathbf{I_p}-\mathbf{A})^\mathbf{T}\mathbf{\Sigma}^{-1}(\mathbf{I_p}-\mathbf{A}))\\
&\qquad-2\mathrm{tr}(\sum_{i=1}^n\mathbf{Y_i}\mathbf{X_i^T}\mathbf{B^T}\mathbf{\Sigma}^{-1}(\mathbf{I_p}-\mathbf{A}))+\mathrm{tr}(\sum_{i=1}^n\mathbf{X_i}\mathbf{X_i^T}\mathbf{B^T}\mathbf{\Sigma}^{-1}\mathbf{B})])\\
=&(2\pi)^{-\frac{np}{2}}.\det(\mathbf{\Sigma})^{-\frac{n}{2}}.|\det(\mathbf{I_p}-\mathbf{A})|^\mathbf{n}.\exp(-\frac{1}{2}[\mathrm{tr}(n.\mathbf{Syy}(\mathbf{I_p}-\mathbf{A})^\mathbf{T}\mathbf{\Sigma}^{-1}(\mathbf{I_p}-\mathbf{A}))\\
&\qquad-2\mathrm{tr}(n.\mathbf{Syx}\mathbf{B^T}\mathbf{\Sigma}^{-1}(\mathbf{I_p}-\mathbf{A}))+\mathrm{tr}(n.\mathbf{Sxx}\mathbf{B^T}\mathbf{\Sigma}^{-1}\mathbf{B})])\\
=&(2\pi)^{-\frac{np}{2}}.\det(\mathbf{\Sigma})^{-\frac{n}{2}}.|\det(\mathbf{I_p}-\mathbf{A})|^\mathbf{n}.\exp(-\frac{1}{2}[n.\mathrm{tr}(\mathbf{Syy}(\mathbf{I_p}-\mathbf{A})^\mathbf{T}\mathbf{\Sigma}^{-1}(\mathbf{I_p}-\mathbf{A}))\\
&\qquad-2n.\mathrm{tr}(\mathbf{Syx}\mathbf{B^T}\mathbf{\Sigma}^{-1}(\mathbf{I_p}-\mathbf{A}))+n.\mathrm{tr}(\mathbf{Sxx}\mathbf{B^T}\mathbf{\Sigma}^{-1}\mathbf{B})])
\end{aligned}
$$

10 Calculation of A−matrix from Beta−matrix

Let $\mathbf{Y_i}=(Y_{i1},\cdots,Y_{ip})^T$ denote the expressions for response variables $1,\cdots,p$, let $\mathbf{X_i}=(X_{i1},\cdots,X_{ik})^T$ be the set of measurements for instrument variables $1,\cdots,k$ and for $i=1,\ldots,n$. For this moment lets assume each response is affected by one and only one instrument i.e. $k=p$ and hence $\mathbf{B}$ is a diagonal matrix. We have the following model:

$$\mathbf{Y_i}=\mathbf{A}\mathbf{Y_i}+\mathbf{B}\mathbf{X_i}+\mathbf{E_i},\quad \mathbf{E_i}\sim N(0,\mathbf{\Sigma})$$

$$\implies (\mathbf{I_p} - \mathbf{A})\mathbf{Y_i} = \mathbf{BX_i} + \mathbf{E_i}$$
$$\implies \mathbf{Y_i} = (\mathbf{I_p} - \mathbf{A})^{-1}\mathbf{BX_i} + (\mathbf{I_p} - \mathbf{A})^{-1}\mathbf{E_i} \tag{10.1}$$

where,

$$\mathbf{A} = \begin{bmatrix} 0 & a_{12} & \dots & a_{1p} \\ a_{21} & 0 & \dots & a_{2p} \\ \vdots & \vdots & \ddots & \vdots \\ a_{p1} & a_{p2} & \dots & 0 \end{bmatrix} \quad \mathbf{B} = \begin{bmatrix} b_1 & 0 & \dots & 0 \\ 0 & b_2 & \dots & 0 \\ \vdots & \vdots & \ddots & \vdots \\ 0 & 0 & \dots & b_p \end{bmatrix}$$

We define

$$\mathbf{Beta} = \begin{bmatrix} \hat{\beta}_{11} & \hat{\beta}_{12} & \dots & \hat{\beta}_{1p} \\ \hat{\beta}_{21} & \hat{\beta}_{22} & \dots & \hat{\beta}_{2p} \\ \vdots & \vdots & \ddots & \vdots \\ \hat{\beta}_{p1} & \hat{\beta}_{p2} & \dots & \hat{\beta}_{pp} \end{bmatrix}$$

where, $\hat{\beta}_{ij}$ denotes the regression coefficient of Y_i on X_j. So, we can assume from equation (10.1),

$$\mathbf{Beta} = (\mathbf{I_p} - \mathbf{A})^{-1}\mathbf{B} \tag{10.2}$$

Claim: We claim that a_{ij} can be calculated from the **Beta** matrix in the following way:

$$a_{ij} = (-1)^{(i-j+1)} \times \frac{\det\left(\begin{bmatrix} \hat{\beta}_{11} & \dots & \hat{\beta}_{1(i-1)} & \hat{\beta}_{1(i+1)} & \dots & \hat{\beta}_{1p} \\ \vdots & \ddots & \vdots & \vdots & \ddots & \vdots \\ \hat{\beta}_{(j-1)1} & \dots & \hat{\beta}_{(j-1)(i-1)} & \hat{\beta}_{(j-1)(i+1)} & \dots & \hat{\beta}_{(j-1)p} \\ \hat{\beta}_{(j+1)1} & \dots & \hat{\beta}_{(j+1)(i-1)} & \hat{\beta}_{(j+1)(i+1)} & \dots & \hat{\beta}_{(j+1)p} \\ \vdots & \ddots & \vdots & \vdots & \ddots & \vdots \\ \hat{\beta}_{p1} & \dots & \hat{\beta}_{p(i-1)} & \hat{\beta}_{p(i+1)} & \dots & \hat{\beta}_{pp} \end{bmatrix}\right)}{\det\left(\begin{bmatrix} \hat{\beta}_{11} & \dots & \hat{\beta}_{1(i-1)} & \hat{\beta}_{1(i+1)} & \dots & \hat{\beta}_{1p} \\ \vdots & \ddots & \vdots & \vdots & \ddots & \vdots \\ \hat{\beta}_{(i-1)1} & \dots & \hat{\beta}_{(i-1)(i-1)} & \hat{\beta}_{(i-1)(i+1)} & \dots & \hat{\beta}_{(i-1)p} \\ \hat{\beta}_{(i+1)1} & \dots & \hat{\beta}_{(i+1)(i-1)} & \hat{\beta}_{(i+1)(i+1)} & \dots & \hat{\beta}_{(i+1)p} \\ \vdots & \ddots & \vdots & \vdots & \ddots & \vdots \\ \hat{\beta}_{p1} & \dots & \hat{\beta}_{p(i-1)} & \hat{\beta}_{p(i+1)} & \dots & \hat{\beta}_{pp} \end{bmatrix}\right)} \tag{10.3}$$

i.e. starting with **Beta** matrix we first remove the i-th column, then from that matrix we first remove the j-th row and calculate the determinant and divide it by the determinant of the matrix calculated by removing the i-th row. The sign of a_{ij} is determined by $(-1)^{i-j+1}$. We will now prove this. Let,

$$(\mathbf{I_p} - \mathbf{A})^{-1} = \begin{bmatrix} M_{11} & M_{12} & \dots & M_{1p} \\ M_{21} & M_{22} & \dots & M_{2p} \\ \vdots & \vdots & \ddots & \vdots \\ M_{p1} & M_{p2} & \dots & M_{pp} \end{bmatrix}$$

From equation (10.2) we have,

$$\mathbf{Beta} = (\mathbf{I_p} - \mathbf{A})^{-1}\mathbf{B}$$

$$\implies \begin{bmatrix} \hat{\beta}_{11} & \hat{\beta}_{12} & \dots & \hat{\beta}_{1p} \\ \hat{\beta}_{21} & \hat{\beta}_{22} & \dots & \hat{\beta}_{2p} \\ \vdots & \vdots & \ddots & \vdots \\ \hat{\beta}_{p1} & \hat{\beta}_{p2} & \dots & \hat{\beta}_{pp} \end{bmatrix} = \begin{bmatrix} M_{11} & M_{12} & \dots & M_{1p} \\ M_{21} & M_{22} & \dots & M_{2p} \\ \vdots & \vdots & \ddots & \vdots \\ M_{p1} & M_{p2} & \dots & M_{pp} \end{bmatrix} \times \begin{bmatrix} b_1 & 0 & \dots & 0 \\ 0 & b_2 & \dots & 0 \\ \vdots & \vdots & \ddots & \vdots \\ 0 & 0 & \dots & b_p \end{bmatrix}$$

$$\implies \hat{\beta}_{ij} = M_{ij}b_j \quad \forall i,j \in \{1,\cdots,p\}$$

Thus replacing $\hat{\beta}_{ij}$ by $M_{ij} \times b_j$ in equation (10.3) we get:

$$a_{ij} = (-1)^{(i-j+1)} \times \frac{\det\left(\begin{bmatrix} M_{11}b_1 & \dots & M_{1(i-1)}b_{i-1} & M_{1(i+1)}b_{i+1} & \dots & M_{1p}b_p \\ \vdots & \ddots & \vdots & \vdots & \ddots & \vdots \\ M_{(j-1)1}b_1 & \dots & M_{(j-1)(i-1)}b_{i-1} & M_{(j-1)(i+1)}b_{i+1} & \dots & M_{(j-1)p}b_p \\ M_{(j+1)1}b_1 & \dots & M_{(j+1)(i-1)}b_{i-1} & M_{(j+1)(i+1)}b_{i+1} & \dots & M_{(j+1)p}b_p \\ \vdots & \ddots & \vdots & \vdots & \ddots & \vdots \\ M_{p1}b_1 & \dots & M_{p(i-1)}b_{i-1} & M_{p(i+1)}b_{i+1} & \dots & M_{pp}b_p \end{bmatrix}\right)}{\det\left(\begin{bmatrix} M_{11}b_1 & \dots & M_{1(i-1)}b_{i-1} & M_{1(i+1)}b_{i+1} & \dots & M_{1p}b_p \\ \vdots & \ddots & \vdots & \vdots & \ddots & \vdots \\ M_{(i-1)1}b_1 & \dots & M_{(i-1)(i-1)}b_{i-1} & M_{(i-1)(i+1)}b_{i+1} & \dots & M_{(i-1)p}b_p \\ M_{(i+1)1}b_1 & \dots & M_{(i+1)(i-1)}b_{i-1} & M_{(i+1)(i+1)}b_{i+1} & \dots & M_{(i+1)p}b_p \\ \vdots & \ddots & \vdots & \vdots & \ddots & \vdots \\ M_{p1}b_1 & \dots & M_{p(i-1)}b_{i-1} & M_{p(i+1)}b_{i+1} & \dots & M_{pp}b_p \end{bmatrix}\right)}$$

$$= (-1)^{(i-j+1)} \times \frac{\det\left(\begin{bmatrix} M_{11} & \dots & M_{1(i-1)} & M_{1(i+1)} & \dots & M_{1p} \\ \vdots & \ddots & \vdots & \vdots & \ddots & \vdots \\ M_{(j-1)1} & \dots & M_{(j-1)(i-1)} & M_{(j-1)(i+1)} & \dots & M_{(j-1)p} \\ M_{(j+1)1} & \dots & M_{(j+1)(i-1)} & M_{(j+1)(i+1)} & \dots & M_{(j+1)p} \\ \vdots & \ddots & \vdots & \vdots & \ddots & \vdots \\ M_{p1} & \dots & M_{p(i-1)} & M_{p(i+1)} & \dots & M_{pp} \end{bmatrix}\right)}{\det\left(\begin{bmatrix} M_{11} & \dots & M_{1(i-1)} & M_{1(i+1)} & \dots & M_{1p} \\ \vdots & \ddots & \vdots & \vdots & \ddots & \vdots \\ M_{(i-1)1} & \dots & M_{(i-1)(i-1)} & M_{(i-1)(i+1)} & \dots & M_{(i-1)p} \\ M_{(i+1)1} & \dots & M_{(i+1)(i-1)} & M_{(i+1)(i+1)} & \dots & M_{(i+1)p} \\ \vdots & \ddots & \vdots & \vdots & \ddots & \vdots \\ M_{p1} & \dots & M_{p(i-1)} & M_{p(i+1)} & \dots & M_{pp} \end{bmatrix}\right)} \tag{10.4}$$

Again, we have,

$$(\mathbf{I_p} - \mathbf{A}) \times (\mathbf{I_p} - \mathbf{A})^{-1} = \mathbf{I_p}$$

$$\implies \begin{bmatrix} 1 & -a_{12} & \dots & -a_{1p} \\ -a_{21} & 1 & \dots & -a_{2p} \\ \vdots & \vdots & \ddots & \vdots \\ -a_{p1} & -a_{p2} & \dots & 1 \end{bmatrix} \times \begin{bmatrix} M_{11} & M_{12} & \dots & M_{1p} \\ M_{21} & M_{22} & \dots & M_{2p} \\ \vdots & \vdots & \ddots & \vdots \\ M_{p1} & M_{p2} & \dots & M_{pp} \end{bmatrix} = \begin{bmatrix} 1 & 0 & \dots & 0 \\ 0 & 1 & \dots & 0 \\ \vdots & \vdots & \ddots & \vdots \\ 0 & 0 & \dots & 1 \end{bmatrix}$$

So, for each $i \in \{1, \cdots, p\}$ there will be p many linear equations involving $a_{i1}, \cdots, a_{i(i-1)}, a_{i(i+1)}, \cdots, a_{ip}$. We have the linear equations as follows:

$$\begin{aligned}
-a_{i1}M_{11} - a_{i2}M_{21} - \cdots + M_{i1} - \cdots - a_{ip}M_{p1} &= 0 \\
-a_{i1}M_{12} - a_{i2}M_{22} - \cdots + M_{i2} - \cdots - a_{ip}M_{p2} &= 0 \\
&\vdots \\
-a_{i1}M_{1i} - a_{i2}M_{2i} - \cdots + M_{ii} - \cdots - a_{ip}M_{pi} &= 1 \\
&\vdots \\
-a_{i1}M_{1p} - a_{i2}M_{2p} - \cdots + M_{ip} - \cdots - a_{ip}M_{pp} &= 0
\end{aligned}$$

So, we have $(p-1)$ many variables $a_{i1}, \cdots, a_{i(i-1)}, a_{i(i+1)}, \cdots, a_{ip}$ and p many equations and we know that solutions for $a_{i1}, \cdots, a_{i(i-1)}, a_{i(i+1)}, \cdots, a_{ip}$ exist which satisfy these p many equations. So, we can take any $(p-1)$ many equations and solve them. Let us take the equations whose RHS is 0. So, we have:

$$\begin{aligned}
-a_{i1}M_{11} - a_{i2}M_{21} - \cdots - a_{ij}M_{j1} - \cdots - a_{ip}M_{p1} &= -M_{i1} \\
-a_{i1}M_{12} - a_{i2}M_{22} - \cdots - a_{ij}M_{j2} - \cdots - a_{ip}M_{p2} &= -M_{i2} \\
&\vdots \\
-a_{i1}M_{1(i-1)} - a_{i2}M_{2(i-1)} - \cdots - a_{ij}M_{j(i-1)} - \cdots - a_{ip}M_{p(i-1)} &= -M_{i(i-1)} \\
-a_{i1}M_{1(i+1)} - a_{i2}M_{2(i+1)} - \cdots - a_{ij}M_{j(i+1)} - \cdots - a_{ip}M_{p(i+1)} &= -M_{i(i+1)} \\
&\vdots \\
-a_{i1}M_{1p} - a_{i2}M_{2p} - \cdots - a_{ij}M_{jp} - \cdots - a_{ip}M_{pp} &= -M_{ip}
\end{aligned}$$

Thus, applying Cramer's rule to solve $(p-1)$ many linear equations in $(p-1)$ many unknowns we have the solutions as follows:

$$a_{ij} = \frac{\det\left(\begin{bmatrix} -M_{11} & \dots & M_{(j-1)1} & -M_{i1} & -M_{(j+1)1} & \dots & -M_{p1} \\ \vdots & \ddots & \vdots & \vdots & \vdots & \ddots & \vdots \\ -M_{1(i-1)} & \dots & M_{(j-1)(i-1)} & -M_{i(i-1)} & -M_{(j+1)(i-1)} & \dots & -M_{p(i-1)} \\ -M_{1(i+1)} & \dots & M_{(j-1)(i+1)} & -M_{i(i+1)} & -M_{(j+1)(i+1)} & \dots & -M_{p(i+1)} \\ \vdots & \ddots & \vdots & \vdots & \vdots & \ddots & \vdots \\ -M_{1p} & \dots & M_{(j-1)p} & -M_{ip} & -M_{(j+1)p} & \dots & -M_{pp} \end{bmatrix}\right)}{\det\left(\begin{bmatrix} -M_{11} & \dots & M_{(j-1)1} & -M_{j1} & -M_{(j+1)1} & \dots & -M_{p1} \\ \vdots & \ddots & \vdots & \vdots & \vdots & \ddots & \vdots \\ -M_{1(i-1)} & \dots & M_{(j-1)(i-1)} & -M_{j(i-1)} & -M_{(j+1)(i-1)} & \dots & -M_{p(i-1)} \\ -M_{1(i+1)} & \dots & M_{(j-1)(i+1)} & -M_{j(i+1)} & -M_{(j+1)(i+1)} & \dots & -M_{p(i+1)} \\ \vdots & \ddots & \vdots & \vdots & \vdots & \ddots & \vdots \\ -M_{1p} & \dots & M_{(j-1)p} & -M_{jp} & -M_{(j+1)p} & \dots & -M_{pp} \end{bmatrix}\right)}$$

$$= \frac{\det\left(\begin{bmatrix} M_{11} & \dots & M_{1(i-1)} & M_{1(i+1)} & \dots & M_{1p} \\ \vdots & \ddots & \vdots & \vdots & \ddots & \vdots \\ M_{(j-1)1} & \dots & M_{(j-1)(i-1)} & M_{(j-1)(i+1)} & \dots & M_{(j-1)p} \\ M_{i1} & \dots & M_{i(i-1)} & M_{i(i+1)} & \dots & M_{ip} \\ M_{(j+1)1} & \dots & M_{(j+1)(i-1)} & M_{(j+1)(i+1)} & \dots & M_{(j+1)p} \\ \vdots & \ddots & \vdots & \vdots & \ddots & \vdots \\ M_{p1} & \dots & M_{p(i-1)} & M_{p(i+1)} & \dots & M_{pp} \end{bmatrix}\right)}{\det\left(\begin{bmatrix} M_{11} & \dots & M_{1(i-1)} & M_{1(i+1)} & \dots & M_{1p} \\ \vdots & \ddots & \vdots & \vdots & \ddots & \vdots \\ M_{(i-1)1} & \dots & M_{(i-1)(i-1)} & M_{(i-1)(i+1)} & \dots & M_{(i-1)p} \\ M_{(i+1)1} & \dots & M_{(i+1)(i-1)} & M_{(i+1)(i+1)} & \dots & M_{(i+1)p} \\ \vdots & \ddots & \vdots & \vdots & \ddots & \vdots \\ M_{p1} & \dots & M_{p(i-1)} & M_{p(i+1)} & \dots & M_{pp} \end{bmatrix}\right)}$$

[Taking transpose of both matrices]

$$= (-1)^{(i-j+1)} \cdot \frac{\det\left(\begin{bmatrix} M_{11} & \dots & M_{1(i-1)} & M_{1(i+1)} & \dots & M_{1p} \\ \vdots & \ddots & \vdots & \vdots & \ddots & \vdots \\ M_{(j-1)1} & \dots & M_{(j-1)(i-1)} & M_{(j-1)(i+1)} & \dots & M_{(j-1)p} \\ M_{(j+1)1} & \dots & M_{(j+1)(i-1)} & M_{(j+1)(i+1)} & \dots & M_{(j+1)p} \\ \vdots & \ddots & \vdots & \vdots & \ddots & \vdots \\ M_{p1} & \dots & M_{p(i-1)} & M_{p(i+1)} & \dots & M_{pp} \end{bmatrix}\right)}{\det\left(\begin{bmatrix} M_{11} & \dots & M_{1(i-1)} & M_{1(i+1)} & \dots & M_{1p} \\ \vdots & \ddots & \vdots & \vdots & \ddots & \vdots \\ M_{(i-1)1} & \dots & M_{(i-1)(i-1)} & M_{(i-1)(i+1)} & \dots & M_{(i-1)p} \\ M_{(i+1)1} & \dots & M_{(i+1)(i-1)} & M_{(i+1)(i+1)} & \dots & M_{(i+1)p} \\ \vdots & \ddots & \vdots & \vdots & \ddots & \vdots \\ M_{p1} & \dots & M_{p(i-1)} & M_{p(i+1)} & \dots & M_{pp} \end{bmatrix}\right)} \tag{10.5}$$

[Moving $M_{i1}, \dots, M_{ip}$ in the numerator matrix from $(j-1)$th row to ith row]

So, we can see that the form of a_{ij} obtained in equation (10.5) is exactly same as in equation (10.4). So, we can say that our claim is correct and we can get $a_{ij}(i \neq j)$ from the **Beta** matrix in the above described manner.

Note: If the number of instruments is greater than the number of response variables, i.e. $k > p$, we will select one valid instrument corresponding to each response variable. Consequently, we will create a new matrix called **Beta_New** by stacking the columns of the **Beta** matrix that correspond to these instrument variables. This can always be done because we assume that each response variable is influenced by at least one valid instrument variable (refer to Section (4)). The **Beta_New** matrix will have dimensions $p \times p$. We will then apply the algorithm described in equation (10.3) to estimate the $\mathbf{A}-$matrix using **Beta_New** matrix. The proof for this approach is exactly similar to the one provided earlier.

11 Calculation of Syx and Syy from Sxx, Beta and SigmaHat

Suppose we have $\mathbf{Y} = (\mathbf{Y_1}, \cdots, \mathbf{Y_p})^T$ and $\mathbf{X} = (\mathbf{X_1}, \cdots, \mathbf{X_k})^T$. $Y_1, \cdots, Y_p$ and $X_1, \cdots, X_k$ are vectors of length n, where n is the umber of observations. Let $\hat{\beta}_{ij}$ i.e. (i, j)th entry of **Beta**, denotes the regression coefficient of $\mathbf{Y_i}$ on $\mathbf{X_j}$, where the regression is performed without including an intercept term. So, we have $\hat{\beta}_{ij}$ as follows:

$$\hat{\beta}_{ij} = \frac{\frac{1}{n}\sum_{t=1}^{n} Y_{it} X_{jt}}{\frac{1}{n}\sum_{t=1}^{n} X_{jt}^2}$$

We have, $\hat{\sigma}_{ij}^2$ i.e. (i, j)th entry of **SigmaHat** as follows:

$$\hat{\sigma}_{ij}^2 = \frac{1}{n}\sum_{t=1}^{n}(Y_{it} - \hat{\beta}_{ij} X_{jt})^2$$

We also know **Sxx**, which is a $k \times k$ matrix, whose (i, j)th entry is given as:

$$(\mathbf{Sxx})_{(i,j)} = \frac{1}{n}\sum_{t=1}^{n} X_{it}X_{jt}$$

First we want to calculate **Syx**, which will be a $p \times k$ matrix, whose (i, j) th entry is given as:

$$(\mathbf{Syx})_{(i,j)} = \frac{1}{n}\sum_{t=1}^{n} Y_{it}X_{jt}$$

We have:

$$\begin{aligned}
&\hat{\beta}_{ij} = (\mathbf{X_j}^T\mathbf{X_j})^{-1}\mathbf{X_j}^T\mathbf{Y_i} \\
\implies &\hat{\beta}_{ij} = \left(\frac{1}{n}\sum_{t=1}^{n} X_{jt}X_{jt}\right)^{-1} \times \left(\frac{1}{n}\sum_{t=1}^{n} Y_{it}X_{jt}\right) \\
\implies &(\mathbf{Syx})_{(i,j)} = \hat{\beta}_{ij} \times (\mathbf{Sxx})_{(j,j)}
\end{aligned}$$

In this way we can calculate the **Syx** matrix. Now, we want to calculate **Syy** matrix, which will be a $p \times p$ matrix, whose (i, j) th entry is given as:

$$(\mathbf{Syy})_{(i,j)} = \frac{1}{n}\sum_{t=1}^{n} Y_{it}Y_{jt}$$

We will first calculate the diagonal entries of **Syy** matrix and then use them to construct the whole matrix. We have,

$$\begin{aligned}
&\hat{\sigma}^2_{ij} = \frac{1}{n}\sum_{t=1}^{n}(Y_{it} - \hat{\beta}_{ij}X_{jt})^2, \quad \forall j \in \{1, \cdots, p\} \\
\implies &\frac{1}{n}\sum_{t=1}^{n} Y^2_{it} = \hat{\sigma}^2_{ij} + 2 \times \hat{\beta}_{ij} \times \left(\frac{1}{n}\sum_{t=1}^{n} Y_{it}X_{jt}\right) - \hat{\beta}^2_{ij}\left(\frac{1}{n}\sum_{t=1}^{n} X^2_{jt}\right), \quad \forall j \in \{1, \cdots, p\} \\
\implies &(\mathbf{Syy})_{(i,i)} = \hat{\sigma}^2_{ij} + 2 \times \hat{\beta}_{ij} \times (\mathbf{Syx})_{(i,j)} - \hat{\beta}^2_{ij} \times (\mathbf{Sxx})_{(j,j)}, \quad \forall j \in \{1, \cdots, p\}
\end{aligned}$$

In this way we can calculate the diagonal entries of **Syy**. We will now try to calculate **Syy**. We have the following equation:

$$\begin{aligned}
&\mathbf{Y_i} = \mathbf{AY_i} + \mathbf{BX_i} + \mathbf{E_i}, \quad i = 1, \ldots, n \\
\implies &(\mathbf{I_p} - \mathbf{A})\mathbf{Y_i} = \mathbf{BX_i} + \mathbf{E_i} \\
\implies &\mathbf{Y_i} = (\mathbf{I_p} - \mathbf{A})^{-\mathbf{1}}\mathbf{BX_i} + (\mathbf{I_p} - \mathbf{A})^{-\mathbf{1}}\mathbf{E_i}, \quad \mathrm{Var}(\mathbf{E_i}) = \mathbf{\Sigma} = \begin{bmatrix} \sigma_1^2 & 0 & \dots & 0 \\ 0 & \sigma_2^2 & \dots & 0 \\ \vdots & \vdots & \ddots & \vdots \\ 0 & 0 & \dots & \sigma_p^2 \end{bmatrix} \\
\implies &\mathbf{E}(\mathbf{YY}^T) = (\mathbf{I_p} - \mathbf{A})^{-1}\mathbf{B} \times \mathbf{E}(\mathbf{XX}^T) \times \mathbf{B}^T(\mathbf{I_p} - \mathbf{A})^{-T} + (\mathbf{I_p} - \mathbf{A})^{-1} \times \mathbf{E}(\mathbf{EE}^T) \times (\mathbf{I_p} - \mathbf{A})^{-T} \\
\implies &\mathbf{Syy} = \mathbf{Beta} \times \mathbf{Sxx} \times \mathbf{Beta}^T + (\mathbf{I_p} - \mathbf{A})^{-1} \times \mathbf{\Sigma} \times (\mathbf{I_p} - \mathbf{A})^{-T} \quad \text{[By SLLN]} \qquad (11.1)
\end{aligned}$$

This is true because earlier we assumed $\mathbf{Beta} = (\mathbf{I_p} - \mathbf{A})^{-1} \times \mathbf{B}$ in equation (10.2). We have also shown in Section (10) that we can estimate $\mathbf{A}$ matrix from $\mathbf{Beta}$ matrix, i.e. we also know $(\mathbf{I_p} - \mathbf{A})^{-1}$ term in equation (11.1). So, the only unknown term in equation (11.1) is $\mathbf{\Sigma}$ matrix. Now we have,

$$\mathbf{Y} = (\mathbf{I_p} - \mathbf{A})^{-1}\mathbf{B}\mathbf{X} + (\mathbf{I_p} - \mathbf{A})^{-1}\mathbf{E}$$
$$\implies \mathbf{Y} = \mathbf{Beta} \times \mathbf{X} + (\mathbf{I_p} - \mathbf{A})^{-\mathbf{1}}\mathbf{E} \tag{11.2}$$

So, if we consider the first row of the equation (11.2) we have,

$$Y_{1i} = \beta_{11}X_{1i} + \cdots + \beta_{1p}X_{pi} + e_{1i}, \quad i = 1, \cdots, n$$

We now have,

$$R_1^2 = 1 - \frac{\sum_{i=1}^n e_{1i}^2}{\sum_{i=1}^n Y_{1i}^2} \tag{11.3}$$

We know $\sum_{i=1}^n Y_{1i}^2$ as this will be the first diagonal entry of $\mathbf{Syy}$. We will now show a way to calculate R_1^2 and from that we will try to calculate $\sum_{i=1}^n e_{1i}^2$. Let r_{0i} denotes the correlation coefficient between $\mathbf{Y_1}$ and $\mathbf{X_i}, i = 1, \cdots, k$ and r_{ij} denotes the correlation coefficients between $\mathbf{X_i}$ and $\mathbf{X_j}$; $i = 1, \cdots, k; j = 1, \cdots, k; i \neq j$. So, we have the following matrix:

$$\mathbf{R} = \begin{bmatrix} 1 & r_{01} & r_{02} & \dots & r_{0p} \\ r_{01} & 1 & r_{12} & \dots & r_{1p} \\ \vdots & \vdots & \vdots & \ddots & \vdots \\ r_{0p} & r_{p1} & r_{p2} & \dots & 1 \end{bmatrix}$$

This matrix can easily be calculated based on $\mathbf{Syx}$ and $\mathbf{Sxx}$. From this $\mathbf{R}$ matrix, R_1^2 can be calculated in the following way:

$$R_1^2 = 1 - \frac{det(\mathbf{R})}{R_{11}} \tag{11.4}$$

where, R_{11} denotes the cofactor of $(1,1)$th element of $\mathbf{R}$. Thus from equation (11.3) and (11.4) we can calculate $\sum_{i=1}^n e_{1i}^2$. We can say that $\frac{1}{n}\sum_{i=1}^n e_{1i}^2$ will be the MLE of the first diagonal element of $(\mathbf{I_p} - \mathbf{A})^{-1} \times \mathbf{\Sigma} \times (\mathbf{I_p} - \mathbf{A})^{-T}$. Thus, in this way we can estimate the diagonal entries of $(\mathbf{I_p} - \mathbf{A})^{-1} \times \mathbf{\Sigma} \times (\mathbf{I_p} - \mathbf{A})^{-T}$. We have shown in Section (10) that we can estimate $\mathbf{A}$ from $\mathbf{Beta}$ matrix. So, let us assume:

$$(\mathbf{I_p} - \mathbf{A})^{-1} = \begin{bmatrix} M_{11} & M_{12} & \dots & M_{1p} \\ M_{21} & M_{22} & \dots & M_{2p} \\ \vdots & \vdots & \ddots & \vdots \\ M_{p1} & M_{p2} & \dots & M_{pp} \end{bmatrix}$$

Thus, equating the diagonal entries of $(\mathbf{I_p} - \mathbf{A})^{-1} \times \mathbf{\Sigma} \times (\mathbf{I_p} - \mathbf{A})^{-T}$ with their MLEs we have:

$$\begin{bmatrix} M_{11}^2 & M_{12}^2 & \dots & M_{1p}^2 \\ M_{21}^2 & M_{22}^2 & \dots & M_{2p}^2 \\ \vdots & \vdots & \ddots & \vdots \\ M_{p1}^2 & M_{p2}^2 & \dots & M_{pp}^2 \end{bmatrix} \times \begin{bmatrix} \sigma_1^2 \\ \sigma_2^2 \\ \vdots \\ \sigma_p^2 \end{bmatrix} = \begin{bmatrix} \frac{1}{n}\sum_{i=1}^n e_{1i}^2 \\ \frac{1}{n}\sum_{i=1}^n e_{2i}^2 \\ \vdots \\ \frac{1}{n}\sum_{i=1}^n e_{pi}^2 \end{bmatrix}$$

$$\implies \begin{bmatrix} \sigma_1^2 \\ \sigma_2^2 \\ \vdots \\ \sigma_p^2 \end{bmatrix} = \begin{bmatrix} M_{11}^2 & M_{12}^2 & \dots & M_{1p}^2 \\ M_{21}^2 & M_{22}^2 & \dots & M_{2p}^2 \\ \vdots & \vdots & \ddots & \vdots \\ M_{p1}^2 & M_{p2}^2 & \dots & M_{pp}^2 \end{bmatrix}^{-1} \times \begin{bmatrix} \frac{1}{n}\sum_{i=1}^n e_{1i}^2 \\ \frac{1}{n}\sum_{i=1}^n e_{2i}^2 \\ \vdots \\ \frac{1}{n}\sum_{i=1}^n e_{pi}^2 \end{bmatrix}$$

As, we have estimated $\mathbf{\Sigma}$ matrix we can now estimate **Syy** from equation (11.2). We have used the idea of Section (10) and (11), to calculate **Syy** and **Syx**, when they are not easily available, from **Sxx**, **Beta** and **SigmaHat**.

12 Details of the Main Simulation results

Utilizing the mathematical model $\mathbf{Y_i} = \mathbf{AY_i} + \mathbf{BX_i} + \mathbf{E_i}$, where $\mathbf{B}$ is set as the identity matrix, we conducted a comprehensive assessment of our algorithm's performance. By generating $\mathbf{X_i}$ and $\mathbf{E_i}$ from a normal distribution, we simulated various scenarios to gauge the effectiveness of our approach. Specifically, we calculated True Positive Rate (TPR), False Positive Rate (FPR), False Discovery Rate (FDR), Matthew's Correlation Coefficient (MCC), and Area Under the Curve (AUC) for **MR.RGM** for both the **Spike and Slab** prior and the **Threshold** prior. These metrics were then compared with those obtained through **OneSampleMR** [3] R package. Our analyses encompassed a range of sample sizes – 10k, 30k, and 50k – as well as different variance explained levels $(1\%, 3\%, 5\%$ and $10\%)$. The study also accounted for network sizes of 5, and 10, network sparsity of $\mathbf{A}$ at 25% and 50%, and response variable interactions of either 0.1 or -0.1. A detailed comparison of these factors is presented in table (2), (3), (4) and (5). Notably, our approach consistently yielded equivalent results when applied to both individual level data and summary level data inputs, further validating its versatility and reliability across different data availability scenarios.

Table 2: Comparison of AUC, TPR, FPR, FDR and MCC between MR.RGM (both with threshold prior and spike and slab prior) and OneSampleMR (for different α) for network size 5 and sparsity 50%.

Sample Size (in k)		10	30	50	10	30	50	10	30	50	10	30	50
Variance explained (in %)		1	1	1	3	3	3	3	5	5	5	10	10
Network size		5	5	5	5	5	5	5	5	5	5	5	5
Sparsity (in %)		50	50	50	50	50	50	50	50	50	50	50	50
AUC	Threshold prior	0.67	0.76	0.81	0.82	0.87	0.89	0.87	0.91	0.92	0.998	0.97	0.97
	Spike & slab prior	0.77	0.86	0.92	0.91	0.99	0.98	0.96	0.98	0.99	0.99	0.99	0.99
	OneSampleMR	0.56	0.67	0.74	0.69	0.76	0.79	0.72	0.78	0.79	0.79	0.79	0.78
Threshold prior	TPR	0.49	0.65	0.75	0.65	0.79	0.83	0.73	0.81	0.85	0.94	0.9	0.93
	FPR	0.16	0.19	0.26	0.12	0.08	0.1	0.05	0.06	0.05	0.01	0.02	0.03
	FDR	0.25	0.21	0.24	0.14	0.10	0.12	0.07	0.08	0.07	0.01	0.03	0.03
	MCC	0.35	0.48	0.51	0.57	0.72	0.74	0.72	0.77	0.81	0.94	0.87	0.90
Spike & slab prior	TPR	0.5	0.62	0.73	0.63	0.92	0.98	0.8	0.97	0.98	0.97	0.99	0.99
	FPR	0.096	0.05	0.04	0.04	0.009	0.006	0.02	0.001	0.002	0.006	0	0
	FDR	0.15	0.07	0.05	0.07	0.009	0.006	0.02	0.001	0.002	0.006	0	0
	MCC	0.44	0.61	0.72	0.62	0.91	0.97	0.8	0.97	0.98	0.96	0.99	0.99
OneSampleMR ($\alpha = 0.025$)	TPR	0.08	0.22	0.39	0.22	0.59	0.76	0.37	0.74	0.80	0.64	0.81	0.81
	FPR	0.04	0.07	0.09	0.05	0.17	0.20	0.11	0.21	0.23	0.16	0.23	0.24
	FDR	0.35	0.23	0.18	0.17	0.22	0.20	0.23	0.22	0.22	0.19	0.22	0.23
	MCC	0.08	0.22	0.35	0.26	0.44	0.57	0.30	0.54	0.58	0.50	0.57	0.57
OneSampleMR ($\alpha = 0.05$)	TPR	0.11	0.33	0.49	0.32	0.66	0.78	0.46	0.77	0.81	0.70	0.81	0.82
	FPR	0.07	0.11	0.14	0.09	0.21	0.24	0.15	0.25	0.25	0.20	0.27	0.28
	FDR	0.40	0.24	0.22	0.21	0.24	0.23	0.25	0.24	0.23	0.22	0.24	0.25
	MCC	0.07	0.26	0.37	0.29	0.46	0.54	0.34	0.52	0.57	0.50	0.55	0.55
OneSampleMR ($\alpha = 0.10$)	TPR	0.20	0.45	0.59	0.43	0.72	0.80	0.58	0.80	0.82	0.77	0.83	0.84
	FPR	0.13	0.19	0.21	0.15	0.28	0.29	0.22	0.30	0.30	0.26	0.32	0.34
	FDR	0.38	0.29	0.26	0.25	0.27	0.26	0.27	0.26	0.26	0.25	0.28	0.28
	MCC	0.10	0.29	0.39	0.31	0.45	0.52	0.37	0.51	0.53	0.52	0.51	0.51

Table 3: Comparison of AUC, TPR, FPR, FDR and MCC between MR.RGM (both with threshold prior and spike and slab prior) and OneSampleMR (for different α) for network size 5 and sparsity 25%.

Sample Size (in k)		10	30	50	10	30	50	10	30	50	10	30	50
Variance explained (in %)		1	1	1	3	3	3	3	5	5	5	10	10
Network size		5	5	5	5	5	5	5	5	5	5	5	5
Sparsity (in %)		25	25	25	25	25	25	25	25	25	25	25	25
AUC	Threshold prior	0.71	0.77	0.76	0.78	0.86	0.86	0.87	0.87	0.90	0.97	0.93	0.94
	Spike & slab prior	0.89	0.94	0.98	0.97	0.99	0.997	0.98	0.99	0.997	0.998	0.998	0.996
	OneSampleMR	0.53	0.57	0.57	0.57	0.60	0.59	0.58	0.61	0.60	0.59	0.62	0.63
Threshold prior	TPR	0.57	0.71	0.74	0.67	0.80	0.83	0.81	0.81	0.85	0.94	0.90	0.90
	FPR	0.20	0.22	0.28	0.13	0.14	0.12	0.08	0.11	0.07	0.02	0.03	0.04
	FDR	0.49	0.45	0.51	0.37	0.34	0.31	0.23	0.26	0.2	0.06	0.1	0.12
	MCC	0.36	0.47	0.44	0.53	0.64	0.67	0.71	0.71	0.79	0.92	0.88	0.85
Spike & slab prior	TPR	0.7	0.84	0.91	0.86	0.97	0.996	0.93	0.99	0.99	0.988	0.998	0.996
	FPR	0.13	0.07	0.03	0.05	0.018	0.004	0.026	0.008	0.002	0.01	0.001	0.001
	FDR	0.34	0.19	0.1	0.15	0.05	0.01	0.07	0.02	0.006	0.01	0.002	0.004
	MCC	0.56	0.76	0.87	0.81	0.95	0.99	0.9	0.98	0.99	0.98	0.98	0.995
OneSampleMR ($\alpha = 0.025$)	TPR	0.07	0.15	0.20	0.14	0.35	0.40	0.22	0.40	0.42	0.35	0.43	0.43
	FPR	0.04	0.08	0.12	0.08	0.19	0.21	0.12	0.21	0.22	0.19	0.23	0.22
	FDR	0.64	0.62	0.65	0.63	0.62	0.61	0.63	0.61	0.61	0.61	0.61	0.61
	MCC	0.08	0.11	0.10	0.09	0.16	0.19	0.12	0.18	0.19	0.16	0.19	0.20
OneSampleMR ($\alpha = 0.05$)	TPR	0.09	0.20	0.27	0.19	0.39	0.42	0.27	0.42	0.44	0.38	0.45	0.46
	FPR	0.07	0.13	0.16	0.13	0.22	0.24	0.16	0.25	0.25	0.22	0.25	0.25
	FDR	0.71	0.63	0.63	0.68	0.63	0.62	0.65	0.62	0.62	0.63	0.62	0.62
	MCC	0.04	0.10	0.13	0.07	0.17	0.18	0.11	0.17	0.18	0.15	0.17	0.20
OneSampleMR ($\alpha = 0.10$)	TPR	0.16	0.27	0.36	0.27	0.42	0.46	0.34	0.47	0.47	0.44	0.49	0.50
	FPR	0.13	0.21	0.23	0.19	0.28	0.28	0.22	0.30	0.29	0.28	0.30	0.29
	FDR	0.69	0.68	0.65	0.67	0.66	0.64	0.66	0.64	0.64	0.65	0.64	0.63
	MCC	0.05	0.07	0.13	0.09	0.13	0.17	0.12	0.16	0.17	0.15	0.17	0.19

Table 4: Comparison of AUC, TPR, FPR, FDR and MCC between MR.RGM (both with threshold prior and spike and slab prior) and OneSampleMR (for different α) for network size 10 and sparsity 50%.

Sample Size (in k)<br>Variance explained (in %)<br>Network size<br>Sparsity (in %)		10<br>1<br>10<br>50	30<br>1<br>10<br>50	50<br>1<br>10<br>50	10<br>3<br>10<br>50	30<br>3<br>10<br>50	50<br>3<br>10<br>50	10<br>3<br>10<br>50	30<br>5<br>10<br>50	50<br>5<br>10<br>50	10<br>5<br>10<br>50	30<br>10<br>10<br>50	50<br>10<br>10<br>50
AUC	Threshold prior	0.66	0.78	0.84	0.83	0.93	0.94	0.91	0.96	0.94	0.98	0.98	0.98
	Spike & slab prior	0.69	0.83	0.9	0.87	0.98	0.98	0.95	0.99	0.99	0.99	0.99	0.99
	OneSampleMR	0.53	0.57	0.59	0.57	0.61	0.61	0.59	0.61	0.62	0.59	0.62	0.61
Threshold prior	TPR	0.65	0.84	0.9	0.78	0.95	0.96	0.86	0.98	0.97	0.96	0.98	0.99
	FPR	0.45	0.58	0.62	0.21	0.27	0.35	0.14	0.17	0.22	0.03	0.04	0.05
	FDR	0.4	0.4	0.39	0.2	0.21	0.25	0.13	0.14	0.17	0.03	0.04	0.05
	MCC	0.21	0.3	0.36	0.57	0.71	0.65	0.73	0.82	0.77	0.92	0.94	0.93
Spike & slab prior	TPR	0.43	0.59	0.7	0.64	0.91	0.96	0.78	0.97	0.98	0.95	0.98	0.98
	FPR	0.13	0.08	0.06	0.06	0.02	0.006	0.03	0.005	0.002	0.01	0.001	0.001
	FDR	0.23	0.12	0.078	0.08	0.02	0.006	0.04	0.005	0.002	0.01	0.002	0.001
	MCC	0.34	0.54	0.67	0.61	0.89	0.96	0.77	0.97	0.98	0.94	0.98	0.98
OneSampleMR ($\alpha = 0.05$)	TPR	0.12	0.27	0.38	0.26	0.54	0.62	0.39	0.62	0.62	0.56	0.66	0.67
	FPR	0.09	0.19	0.26	0.19	0.35	0.41	0.26	0.41	0.44	0.37	0.45	0.46
	FDR	0.44	0.41	0.40	0.41	0.39	0.40	0.40	0.39	0.40	0.40	0.40	0.41
	MCC	0.04	0.10	0.14	0.09	0.20	0.21	0.14	0.22	0.21	0.19	0.22	0.21
OneSampleMR ($\alpha = 0.10$)	TPR	0.20	0.36	0.48	0.36	0.60	0.65	0.47	0.66	0.68	0.61	0.69	0.70
	FPR	0.16	0.25	0.34	0.26	0.41	0.46	0.34	0.45	0.49	0.43	0.49	0.52
	FDR	0.45	0.41	0.41	0.42	0.40	0.41	0.41	0.41	0.42	0.41	0.42	0.42
	MCC	0.05	0.12	0.15	0.10	0.19	0.20	0.14	0.20	0.20	0.18	0.20	0.20
OneSampleMR ($\alpha = 0.15$)	TPR	0.26	0.44	0.54	0.43	0.64	0.68	0.54	0.69	0.71	0.64	0.71	0.72
	FPR	0.22	0.32	0.39	0.33	0.45	0.50	0.39	0.49	0.52	0.47	0.53	0.56
	FDR	0.46	0.42	0.42	0.43	0.41	0.42	0.42	0.42	0.42	0.42	0.43	0.44
	MCC	0.05	0.12	0.15	0.11	0.19	0.19	0.14	0.19	0.20	0.18	0.19	0.18

Table 5: Comparison of AUC, TPR, FPR, FDR and MCC between MR.RGM (both with threshold prior and spike and slab prior) and OneSampleMR (for different α) for network size 10 and sparsity 25%.

Sample Size(in k)<br>Variance explained(in %)<br>Network size<br>Sparsity(in %)		10<br>1<br>10<br>25	30<br>1<br>10<br>25	50<br>1<br>10<br>25	10<br>3<br>10<br>25	30<br>3<br>10<br>25	50<br>3<br>10<br>25	10<br>3<br>10<br>25	30<br>5<br>10<br>25	50<br>5<br>10<br>25	10<br>5<br>10<br>25	30<br>10<br>10<br>25	50<br>10<br>10<br>25
AUC	Threshold prior	0.74	0.83	0.84	0.87	0.92	0.9	0.93	0.95	0.93	0.98	0.99	0.98
	Spike & slab prior	0.87	0.94	0.97	0.96	0.99	0.99	0.98	0.995	0.99	0.99	0.994	0.995
	OneSampleMR	0.52	0.49	0.49	0.50	0.49	0.49	0.51	0.50	0.49	0.49	0.49	0.48
Threshold prior	TPR	0.7	0.87	0.89	0.84	0.95	0.93	0.91	0.98	0.94	0.98	0.99	0.98
	FPR	0.33	0.42	0.45	0.16	0.23	0.25	0.1	0.12	0.16	0.02	0.04	0.05
	FDR	0.34	0.41	0.4	0.62	0.64	0.61	0.77	0.79	0.7	0.94	0.93	0.89
	MCC	0.56	0.56	0.57	0.35	0.4	0.4	0.23	0.26	0.33	0.07	0.09	0.1
Spike & slab prior	TPR	0.69	0.8	0.88	0.83	0.96	0.99	0.9	0.99	0.99	0.98	0.99	0.99
	FPR	0.11	0.07	0.04	0.05	0.013	0.006	0.03	0.004	0.002	0.009	0.001	0.001
	FDR	0.31	0.19	0.11	0.14	0.04	0.017	0.08	0.01	0.008	0.02	0.004	0.004
	MCC	0.58	0.73	0.84	0.79	0.95	0.98	0.88	0.99	0.99	0.97	0.99	0.99
OneSampleMR ($\alpha = 0.05$)	TPR	0.07	0.13	0.19	0.14	0.25	0.29	0.20	0.29	0.30	0.26	0.30	0.31
	FPR	0.08	0.14	0.20	0.14	0.26	0.29	0.19	0.29	0.31	0.26	0.31	0.33
	FDR	0.75	0.77	0.75	0.75	0.75	0.75	0.74	0.75	0.75	0.75	0.75	0.75
	MCC	0.01	0.02	0.01	0.00	0.01	0.01	0.00	0.00	0.01	0.00	0.00	0.01
OneSampleMR ($\alpha = 0.10$)	TPR	0.14	0.20	0.26	0.21	0.30	0.33	0.27	0.33	0.34	0.32	0.34	0.35
	FPR	0.13	0.22	0.27	0.21	0.31	0.34	0.26	0.34	0.36	0.32	0.35	0.37
	FDR	0.73	0.76	0.75	0.75	0.75	0.75	0.74	0.75	0.75	0.75	0.75	0.76
	MCC	0.01	0.02	0.00	0.00	0.01	0.01	0.01	0.00	0.02	0.00	0.02	0.03
OneSampleMR ($\alpha = 0.15$)	TPR	0.21	0.26	0.32	0.27	0.35	0.37	0.32	0.37	0.38	0.36	0.38	0.39
	FPR	0.19	0.28	0.32	0.27	0.36	0.39	0.31	0.38	0.40	0.36	0.40	0.42
	FDR	0.72	0.76	0.75	0.74	0.75	0.75	0.73	0.75	0.75	0.75	0.75	0.76
	MCC	0.02	0.02	0.00	0.00	0.00	0.01	0.02	0.01	0.02	0.00	0.02	0.03

13 Selection of Instrumental Variables and Real Data Application

13.1 Selection of Instrumental Variables in Real Gene Expression Data

In MR studies, selecting appropriate instrumental variables (IVs) is crucial for obtaining reliable estimates. For researchers applying the **MR.RGM** method to real gene expression data, the choice of IVs significantly impacts the validity and precision of causal estimates. Below are key

recommendations for selecting IVs in the context of gene expression studies:

1. **cis-eQTL Selection:** In gene expression studies, cis-eQTLs (expression quantitative trait loci) are SNPs located within a short distance from the gene of interest (typically within 1 Mb). These SNPs influence the gene's expression levels and are well-suited as natural instruments. By focusing on cis-eQTLs, researchers ensure that the IVs are biologically relevant and specific to the gene under study. This approach leverages the natural variation in gene expression to infer causal effects.

2. **Top K SNPs as IVs:** It is advisable to select the top K cis-eQTLs (usually 5 or 10) based on their association strength with gene expression levels. This can be achieved by ranking SNPs according to their p-values or effect sizes from the cis-eQTL analysis. Selecting a manageable number of top SNPs helps in reducing dimensionality while retaining the most impactful IVs, thus simplifying the model without sacrificing essential information. This method is illustrated in the later real data application section.

3. **Model Identifiability:** Ensuring that each gene has at least one unique IV—one not shared with other genes—is crucial for model identifiability.

4. **Effect of Instrument Selection:** The choice of strong and valid IVs significantly impacts the accuracy and precision of causal estimates. Weak instruments can lead to biased or imprecise results. Therefore, selecting a reasonable number (e.g., 5 or 10) of top SNPs or top PCs is recommended. Empirical evidence from simulations shows that choosing a limited number of top cis-eQTLs per gene yields reliable and robust results.

The implementation of these IV selection strategies in real data, along with a practical example, is presented in the following section.

13.2 Real Data Application

We applied **MR.RGM** to a real gene expression dataset from the GTEx [4] Analysis V7 database, which offers a wealth of information on human gene expression across various tissues. Specifically, we analyzed the muscle skeletal tissues from 332 donors whose gene expression and SNPs are both available.

For illustration, we selected the following 15 genes: `"TWIST1", "FGFBP3", "ITM2C", "FGF12", "ASPSCR1", "TMEM230", "NUDT4", "VPS25", "PAN3", "RSRC1", "PTPN21", "CCT6A", "C1orf56", "PPIL2", and "DUSP18"`. Through cis-eQTL analysis, we extracted the top 15 SNPs for each gene. Some SNPs were overlapped, leading to a total of 31 unique variants.

We ran **MR.RGM** with **nIter** $= 50,000$, **nBurnin** $= 10,000$, and **Thin** $= 10$. The runtime was 32.329 seconds. Based on the estimated **GammaEst**, representing the edge inclusion probabilities, we applied thresholds of 0.5 and 0.75 to obtain the gene networks (Figure (11)). This approach demonstrates how to construct the input data and apply the **RGM** methodology effectively.

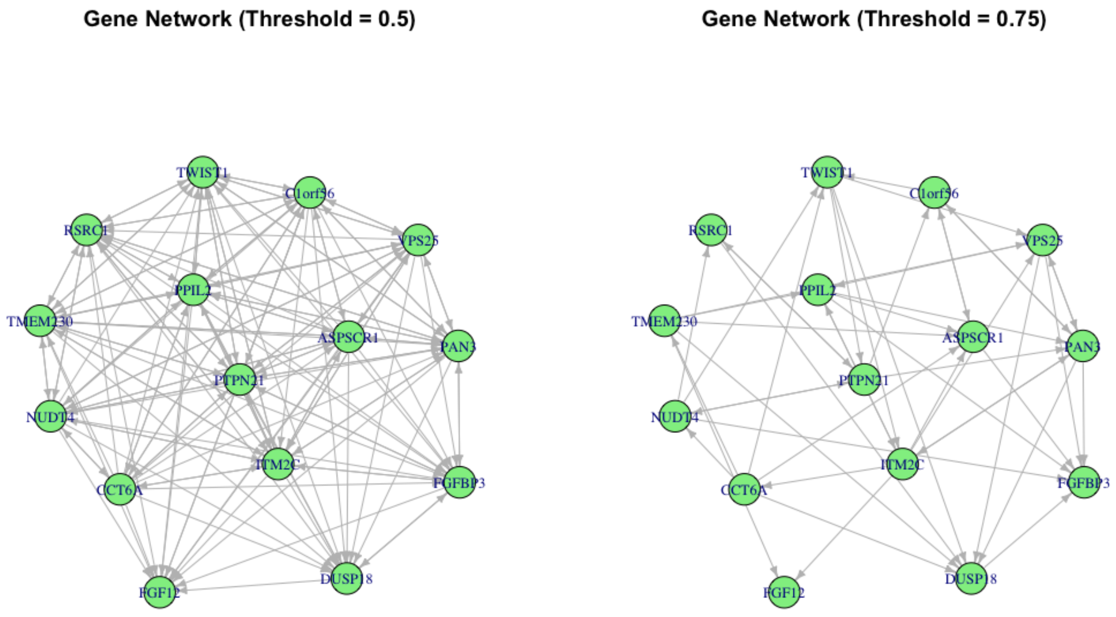

Figure 11: Gene networks constructed using the **MR.RGM** method with varying thresholds. The left plot shows the network with a threshold of 0.5, while the right plot depicts the network with a threshold of 0.75.

References

[1] Bitan Sarkar and Yang Ni. MR.RGM: Multivariate Bidirectional Mendelian Randomization Networks, 2024. R package version 0.0.3.

[2] Yang Ni, Yuan Ji, and Peter Müller. Reciprocal graphical models for integrative gene regulatory network analysis. 2018.

[3] Tom Palmer, Wes Spiller, and Eleanor Sanderson. OneSampleMR: One Sample Mendelian Randomization and Instrumental Variable Analyses, 2023. R package version 0.1.3.

[4] GTEx Consortium. The gtex consortium atlas of genetic regulatory effects across human tissues. Science, 369(6509):1318–1330, 2020.
